# Review of Afrotropical *Cryptopimpla* Taschenberg (Hymenoptera, Ichneumonidae, Banchinae), with description of nine new species

**DOI:** 10.3897/zookeys.640.10334

**Published:** 2016-12-13

**Authors:** Terry Reynolds Berry, Simon van Noort

**Affiliations:** 1 Department of Natural History, Iziko Museums of South Africa, PO Box 61, Cape Town, 8000, South Africa; 2 Stellenbosch University, Department of Botany and Zoology, Evolutionary Genomics Group, Private Bag X1, Stellenbosch 7602, South Africa; 3 Department of Biological Sciences, University of Cape Town, Private Bag, Rondebosch, 7701, South Africa

**Keywords:** Afrotropical region, Hymenoptera, Ichneumonoidea, Lucid identification keys, Atrophini, rubrithorax species-group, goci species-group, South Africa, systematics, taxonomy

## Abstract

The Afrotropical banchine fauna (Hymenoptera: Ichneumonidae) comprises 12 genera. One of these, *Cryptopimpla* Taschenberg, 1863, is a predominately northern hemisphere genus represented by 47 described species of which only one is known from the Afrotropical region. We describe nine new species of this rare Afrotropical genus: *Cryptopimpla elongatus* **sp. n.**, *Cryptopimpla fernkloofensis* **sp. n.**, *Cryptopimpla goci* **sp. n.**, *Cryptopimpla hantami* **sp. n.**, *Cryptopimpla kogelbergensis* **sp. n.**, *Cryptopimpla neili* **sp. n.**, *Cryptopimpla onyxi* **sp. n.**, *Cryptopimpla parslactis* **sp. n.**, and *Cryptopimpla zwarti* **sp. n.** All the Afrotropical species are only known from South Africa. Online interactive Lucid keys to the nine *Cryptopimpla* species are available at: http://www.waspweb.org.

## Introduction

The Afrotropical Banchinae are represented by 12 genera: *Apophua* Morley, *Atropatopsis* Sudheer & Narendran, *Atropha* Kriechbaumer, *Cryptopimpla* Taschenberg, *Exetastes* Gravenhorst, *Glyptopimpla* Morley, *Himertosoma* Schmiedeknecht, *Lissonota* Gravenhorst, *Sjostedtiella* Szépligeti, *Spilopimpla* Cameron, *Syzeuctus* Förster, and *Tossinola* Viktorov (Yu et al. 2012). *Cryptopimpla* belongs to the tribe Atrophini and is a predominately northern hemisphere genus represented by 47 described species, with highest species richness in the temperate regions (Yu et al. 2012; Sheng and Zheng 2005; Kuslitzky 2007; Sheng 2011; Takasuka et al. 2011). A single South African species, *Cryptopimpla rubrithorax* Morley is known from the Afrotropical region (Yu et al. 2012). The genus *Crytopimpla* was defined by Townes (1969) and by Chandra and Gupta (1977).

Over the last 25 years, the temperate winter rainfall region of South Africa encompassing the Cape Floral Kingdom has been fairly extensively sampled by the second author, and large numbers of ichneumonids, including new species of *Cryptopimpla*, have been collected from previously poorly sampled habitats. Sampling inventories have also been conducted in the summer rainfall region of South Africa as well as in other African countries including Central African Republic, Gabon, Kenya, Namibia, Tanzania and Uganda. However, no *Cryptopimpla* species were recorded from these surveys. To our knowledge there are no additional specimens present in historical world collections. In this paper, we describe nine new species from South Africa and provide interactive Lucid identification keys that are available online at http://www.waspweb.org.

## Material and methods

The new species were compared and analyzed in the context of the Afrotropical fauna. Where possible species delimitation was assessed in a world context, but many of the descriptions of species from other regions are not comprehensive in their character assessment. These types were not examined and hence strict comparison to species from other regions was not performed, but this should not compromise the taxonomic results since almost all species of *Cryptopimpla* are exclusive of a single biogeographic region (Yu et al. 2012).

### Photographs

Specimens were point mounted on black, acid-free cards for examination (using a Leica M205C stereomicroscope with LED light source), photography and long-term preservation. Images were acquired using either the EntoVision® multiple-focus imaging system or the Leica LAS 4.4 imaging system. The EntoVision® system comprised a Leica® M16 microscope with a JVC® KY–75U 3–CCD digital video camera attached that fed image data to a notebook computer. The program Cartograph® 5.6.0 was used to manage image acquisition using an automated Z-stepper and merging of the image series into a single in-focus image. The Leica LAS 4.4 system comprised a Leica® Z16 microscope with a Leica DFC450 Camera with a 0.63× video objective attached. Leica Application Suite V 4.4 software was installed on a desk top computer. Lighting was achieved using techniques summarized in Buffington et al. (2005), Kerr et al. (2008) and Buffington and Gates (2009). All images presented in this paper are available at http://www.waspweb.org.

### Depositories

BMNH
 The Natural History Museum, London, England (Gavin Broad) 


SAMC
 Iziko South African Museum, Cape Town, South Africa (Simon van Noort) 


### Nomenclature and abbreviations

The morphological terminology mainly follows Wahl and Sharkey (1993), and the wing venation nomenclature follows Gauld (1991). Most morphological terms are also defined on the HymAToL website (http://www.hymatol.org) and HAO website (http://portal.hymao.org/projects/32/public/ontology/). The following morphometric abbreviations are used (in order of appearance in the descriptions):

Body length: from toruli to metasomal apex (mm).

Antenna length: from base of scape to flagellar apex (mm).

Fore wing length: from anterior end of tegula to wing apex (mm).

CT (clypeal transversality index): maximum width of clypeus : length between base of tentorial pit to apex of clypeal edge.

ML (malar line index): shortest distance between eye and mandible : basal width of mandible.

IO (inter-ocellar index): shortest distance between posterior ocelli : ocellus diameter.

OO (oculo-ocellar index): shortest distance between eye and posterior ocellus : ocellus diameter.

Fl1 (length index of flagellomere 1): length : width of flagellomere 1.

OT (ovipositor sheath-hind tibia index): length of ovipositor sheath : length of hind tibia.

The first three measurements (an absolute measure) were measured on all specimens in the type series, with measurements from the primary type reported separately in brackets if necessary.

## Results

### Cryptopimpla

Taxon classificationAnimaliaHymenopteraIchneumonidae

Taschenberg, 1863

Cryptopimpla Taschenberg, 1893. Zeitschrift für die Gesammten Naturwissenschaften, 21:292. Type-species Phytodietus blandus Gravenhorst, 1914.

#### Diagnosis

(updated from Townes 1969, Sheng 2011 and Takasuka et al. 2011). Clypeus small, convex and may have a curved lip on the ventral margin. Occipital carina complete; joining hypostomal carina above base of mandible. Upper tooth of mandible longer than the lower tooth. Apical 0.3–0.4 of flagellum tapered to a slender apex. Pronotum without epomia. Lower half of mesopleuron weakly convex or flat. Hind edge of metanotum with projection absent to well-defined. Posterior transverse carina of propodeum present or absent; pleural carina present or absent; propodeal spiracle round or almost so. Fore wing with areolet present, anteriorly truncate or with short petiole; vein 2m-cu with two closely spaced bullae, or with a single bulla 0.5 to 1.0 times as long as the section of 2m-cu below the bulla. Hind wing with first abscissa of Cu1 weakly reclivous; distal abscissa of Cu1 meeting cu-a distinctly closer to M than to 1A. First tergum evenly and strongly tapered toward base, with a glymma, spiracle anterior to middle of tergum, surface matt to subpolished, with sparse or irregular medium-sized punctures and often some wrinkling; dorsal profile of tergum 1 moderately to strongly convex near base and weakly convex near apex; median dorsal carina absent. Apical portion of metasoma weakly to strongly compressed. Ovipositor sheath approximately 0.6 times as long as hind tibia. Ovipositor straight, sometimes upcurved, its subapical portion with a dorsal notch.

### Species-groups

The Afrotropical species cluster in two morphological species-groups:

*rubrithorax* species-group (*Cryptopimpla elongatus*, *Cryptopimpla fernkloofensis*, *Cryptopimpla hantami*, *Cryptopimpla neili*, *Cryptopimpla onyxi*, *Cryptopimpla parslactis*, *Cryptopimpla rubrithorax*, and *Cryptopimpla zwarti*) is defined by the presence of a weakly convex clypeus with a curved lip on the ventral margin, small tentorial pits, absence of the pleural carinae, and absence of the posterior transverse carina on the propodeum.

*goci* species-group (*Cryptopimpla goci* and *Cryptopimpla kogelbergensis*) is defined by the presence of a convex and bulbous clypeus with large tentorial pits, pleural carinae, and a distinct and well-defined posterior transverse carina on propodeum.

#### Key to Afrotropical species of the genus *Cryptopimpla*

**Table:** 

	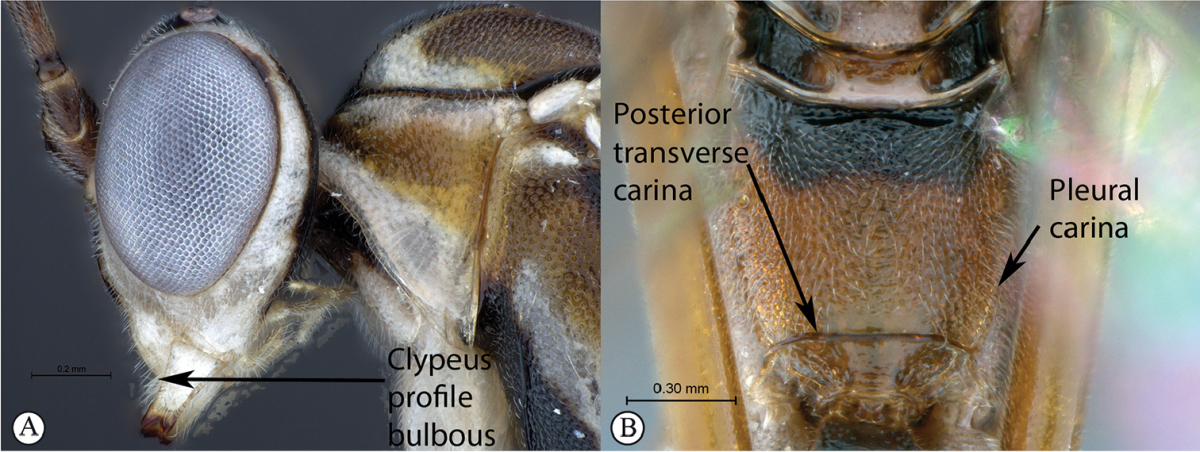	
1	Clypeal profile distinctly convex and bulbous (A). Pleural carinae of propodeum present, but may be weak; posterior transverse carina present and well-defined (B)	**2**
	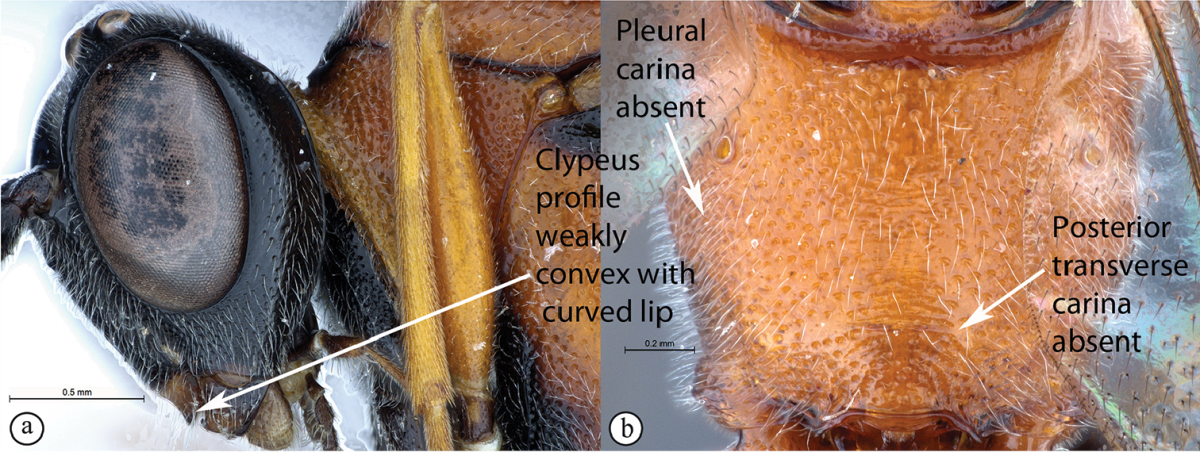	
–	Clypeal profile weakly convex with a curved lip on ventral margin (a). Pleural carinae and posterior transverse carina of propodeum absent (b)	**3**
	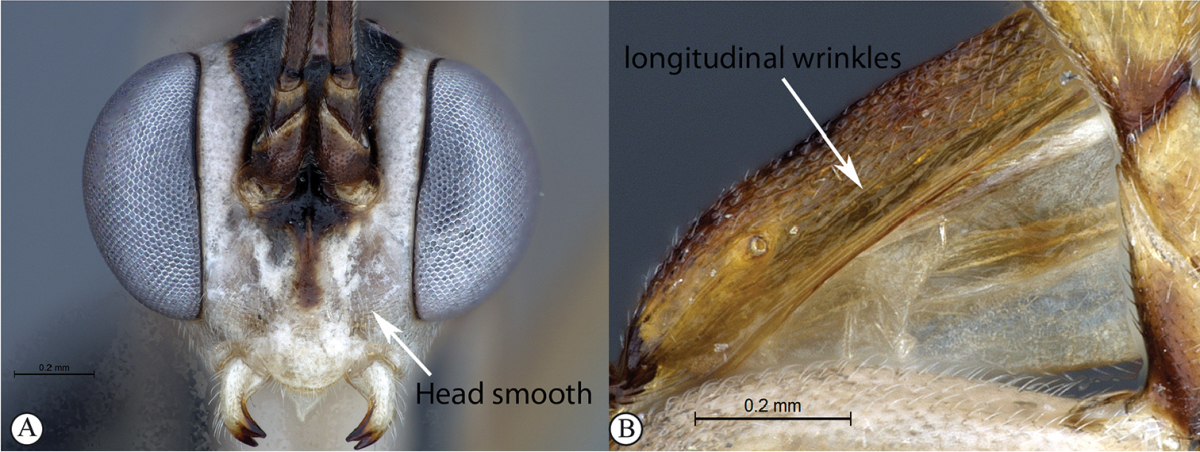	
2	Head smooth, impunctate (A). Dorsolateral carinae on tergum 1 substituted with longitudinal wrinkles (B)	***Cryptopimpla kogelbergensis* sp. n.**
	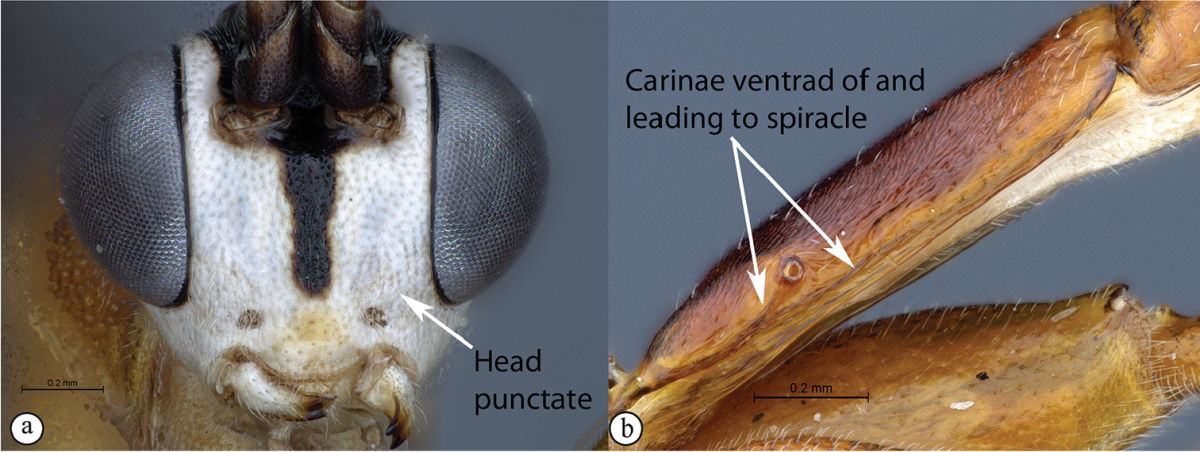	
–	Head finely punctate (a). Dorsolateral carinae on tergum 1 present as a carina ventrad of spiracle, with a secondary carina leading from the ventral carina to the spiracle (b)	***Cryptopimpla goci* sp. n.**
	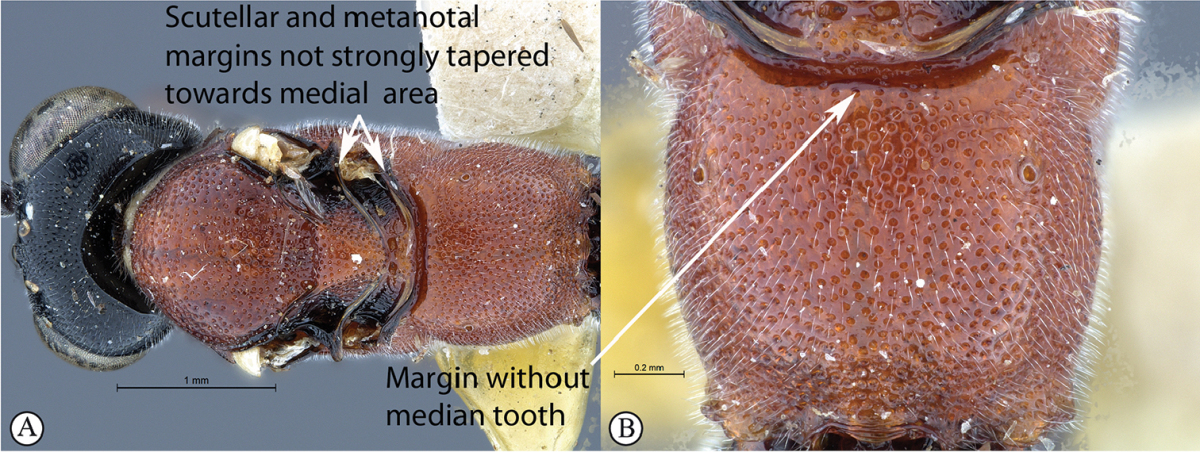	
3	Mesosoma with scutellar and metanotal margins not strongly tapered towards medial area (A). Propodeal anterior margin without defined medial tooth, but may have a blunt medial projection (B)	**4**
	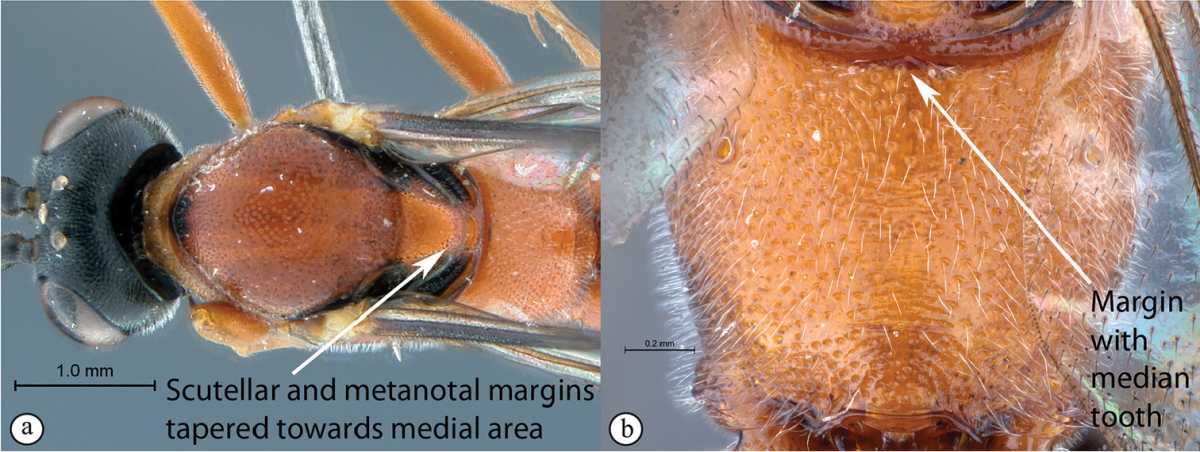	
–	Mesosoma with scutellar margin and metanotal margin tapered towards medial area (a). Propodeal anterior margin with medial tooth (b)	***Cryptopimpla fernkloofensis* sp. n.**
	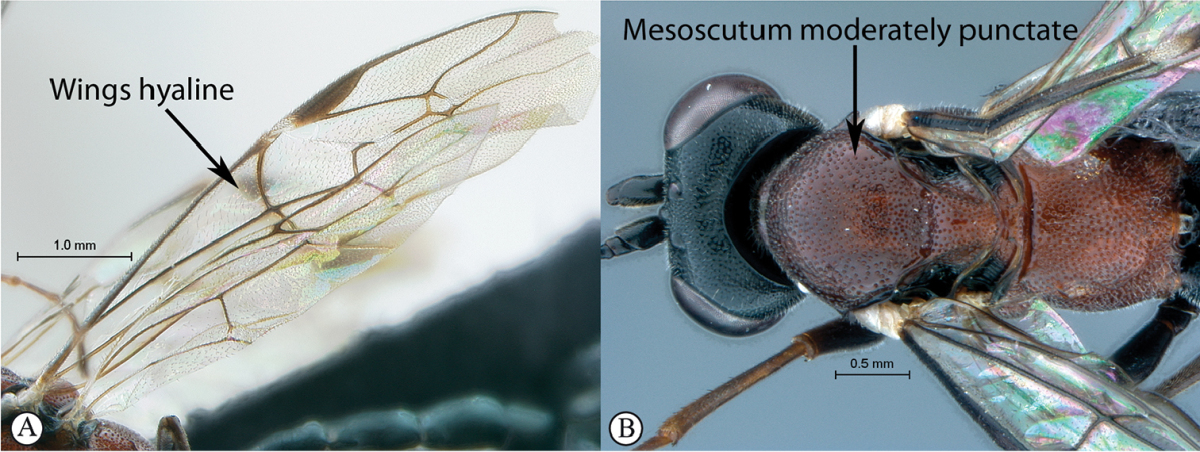	
4	Wings hyaline (A). Mesoscutum moderately punctate (B)	**5**
	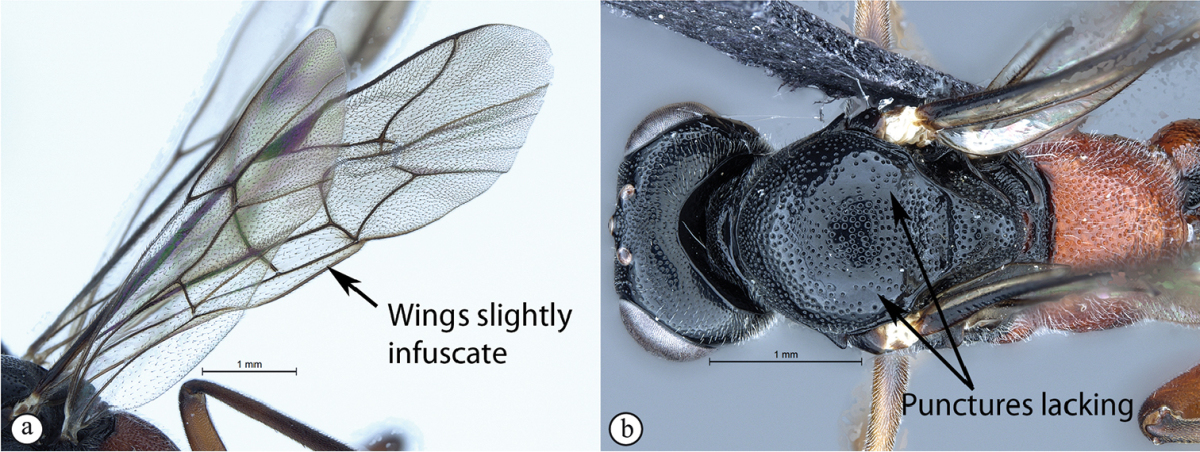	
–	Wings slightly infuscate, venation darker (a). Mesoscutum with fewer punctures inward of wing bases (b)	***Cryptopimpla parslactis* sp. n.**
	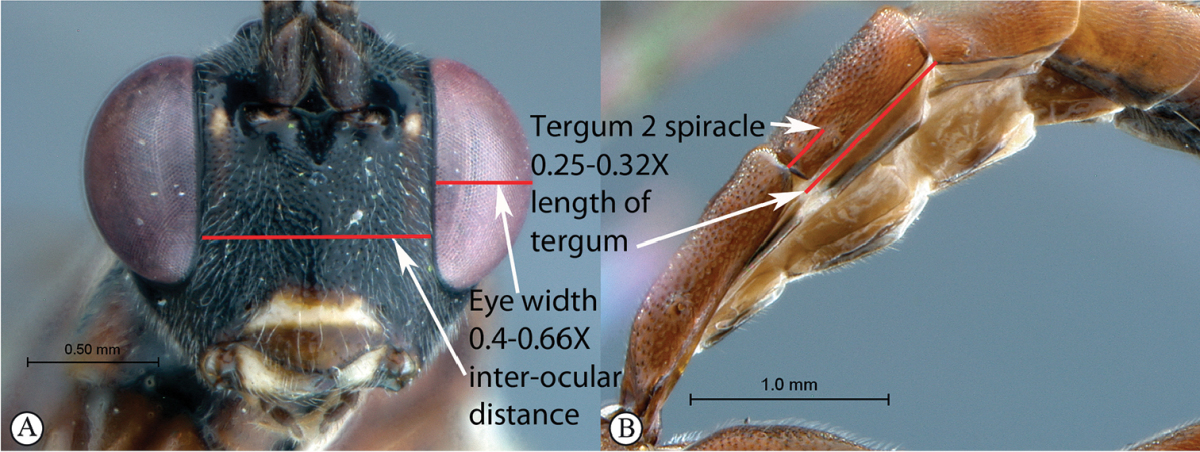	
5	Eye in anterior view narrow to moderately-sized: eye maximum width in anterior view 0.4–0.66 times shortest inter-ocular distance (A). Spiracle of tergum 2 situated at basal 0.25–0.32 of tergum (B)	**6**
	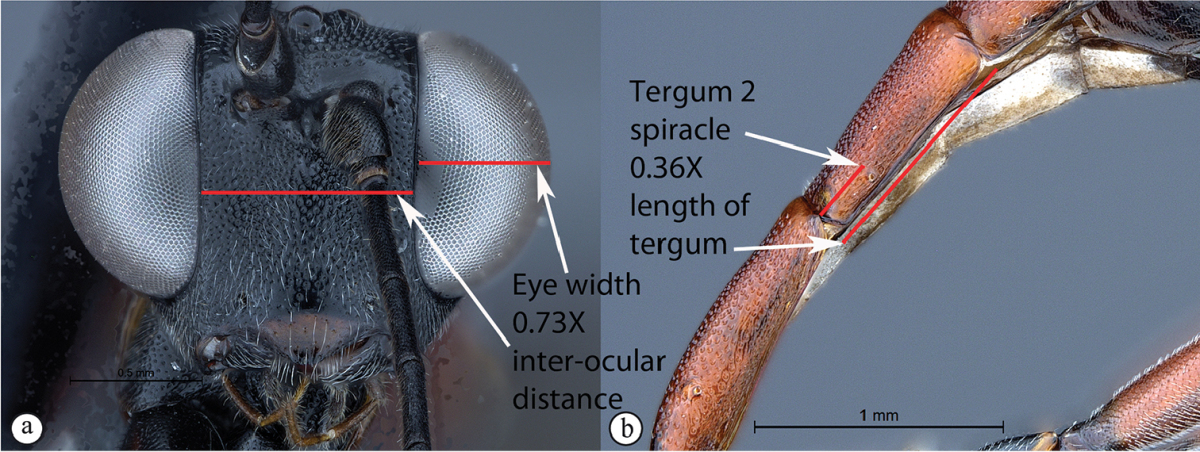	
–	Eye in anterior view larger, bulbous: eye maximum width in anterior view 0.73 times shortest inter-ocular distance (a). Spiracle of tergum 2 situated at basal 0.36 of tergum (b)	***Cryptopimpla elongatus* sp. n.**
	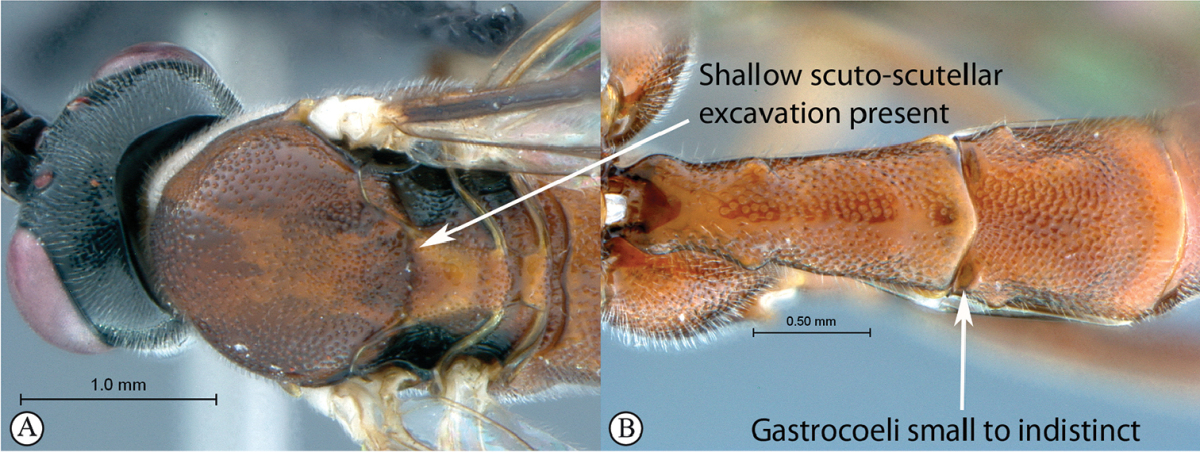	
6	Scuto-scutellar excavation shallow, without deep indentations laterally (a). Metasomal tergum 2 with gastrocoeli small to indistinct (b)	**7**
	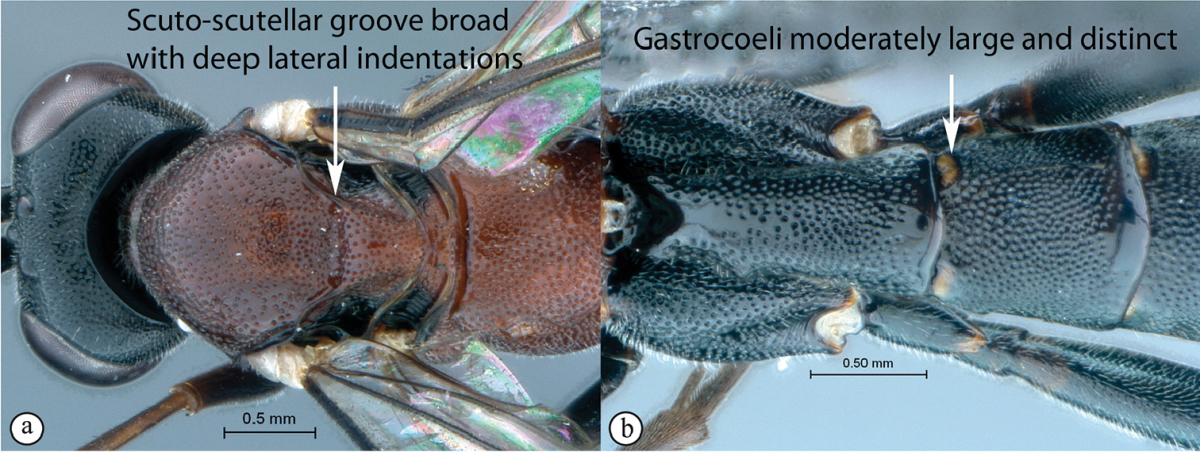	
–	Scuto-scutellar groove broad with deep lateral indentations (A). Metasomal tergum 1 with gastrocoeli moderately large and distinct (B)	**8**
	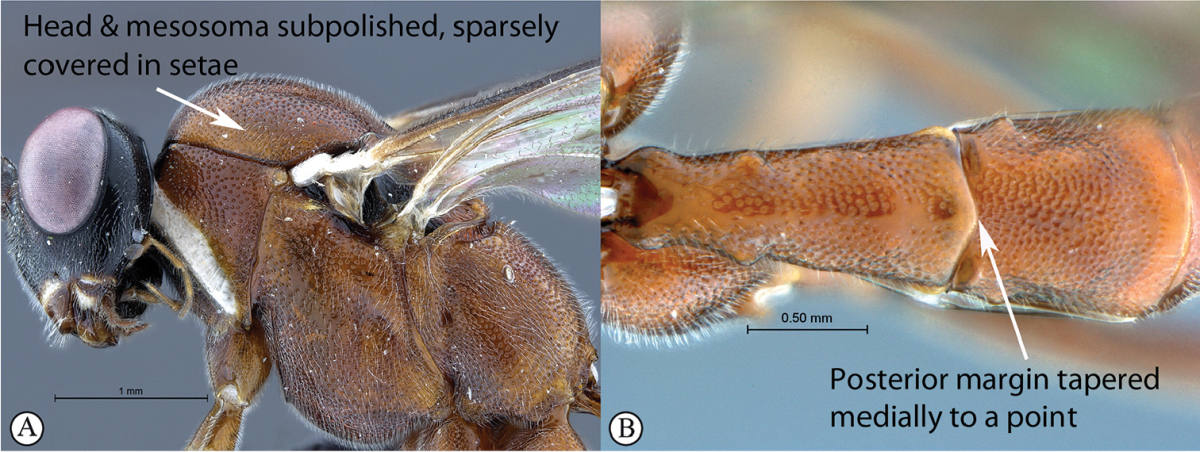	
7	Head and mesosoma subpolished, sparsely covered in short setae (A). Metasomal tergum 1 with posterior margin medially tapered to a point (B)	***Cryptopimpla neili* sp. n.**
	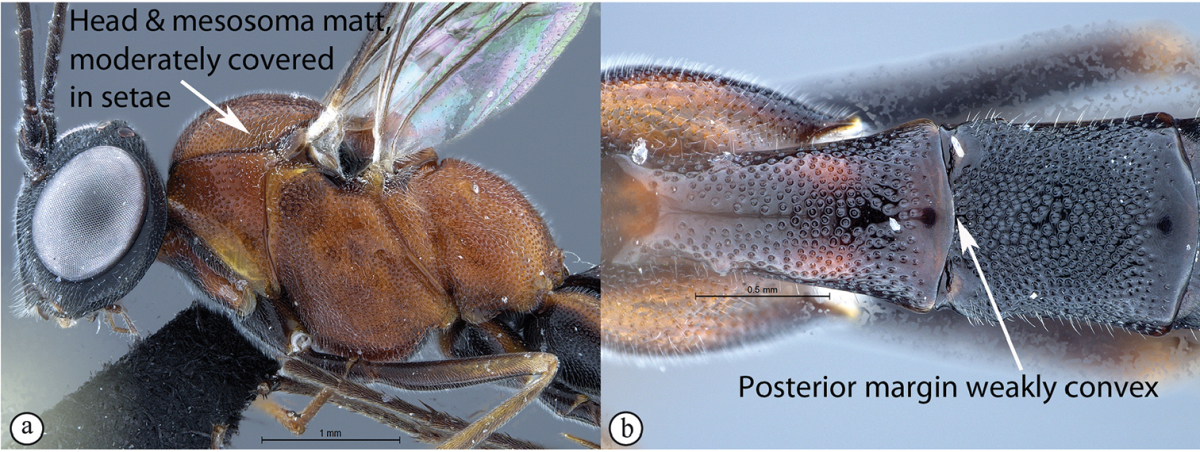	
–	Head and mesosoma matt, moderately covered in short setae (a). Metasomal tergum 1 with posterior margin weakly convex (b)	***Cryptopimpla hantami* sp. n.**
	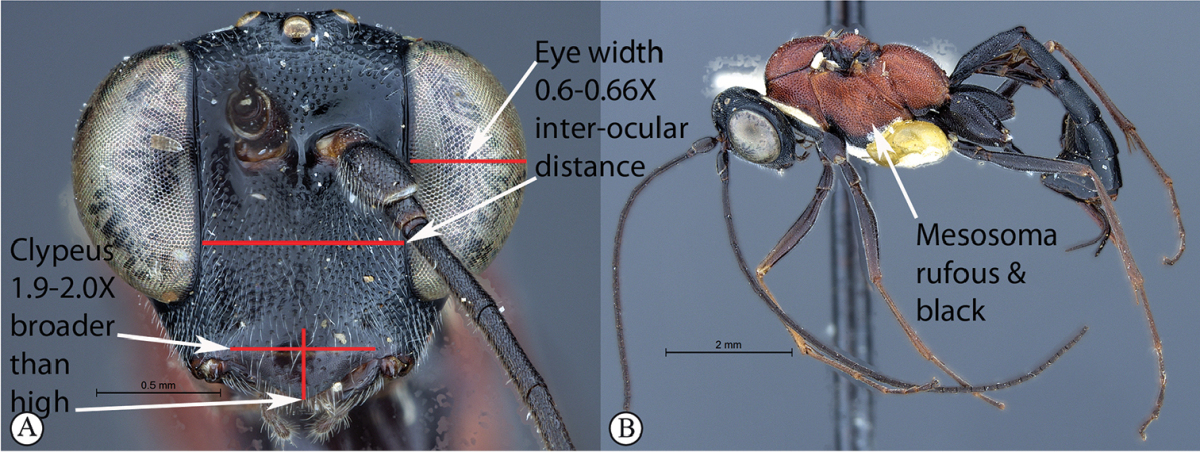	
8	Eye maximum width in anterior view 0.6–0.66 times shortest inter-ocular distance (B). Clypeus 1.9–2 times broader than high. Mesosoma predominantly rufous with black markings (C)	**9**
	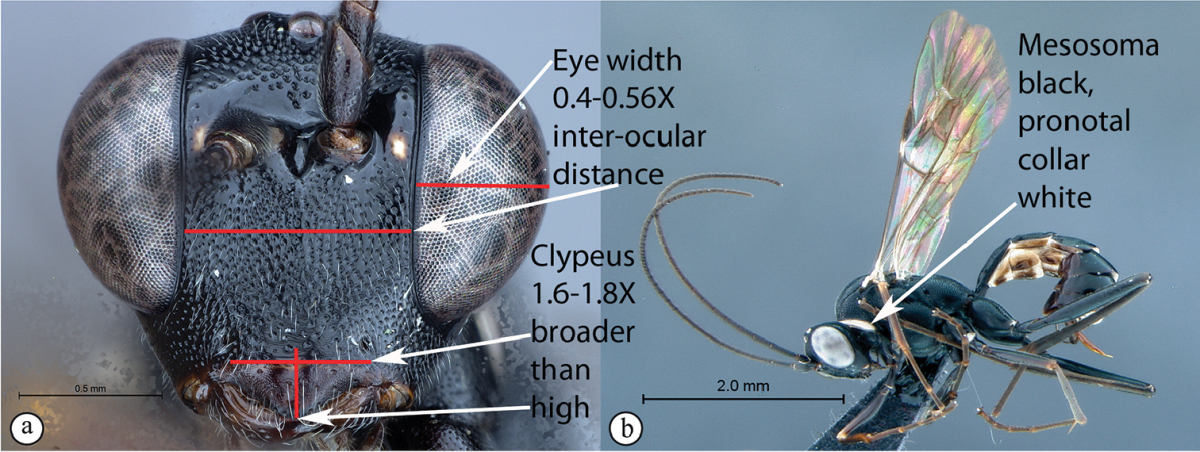	
–	Eye narrower, its maximum width in anterior view 0.4–0.56 times shortest inter-ocular distance (B). Clypeus 1.6–1.8 times broader than high. Mesosoma black with white pronotal collar (C)	***Cryptopimpla onyxi* sp. n.**
	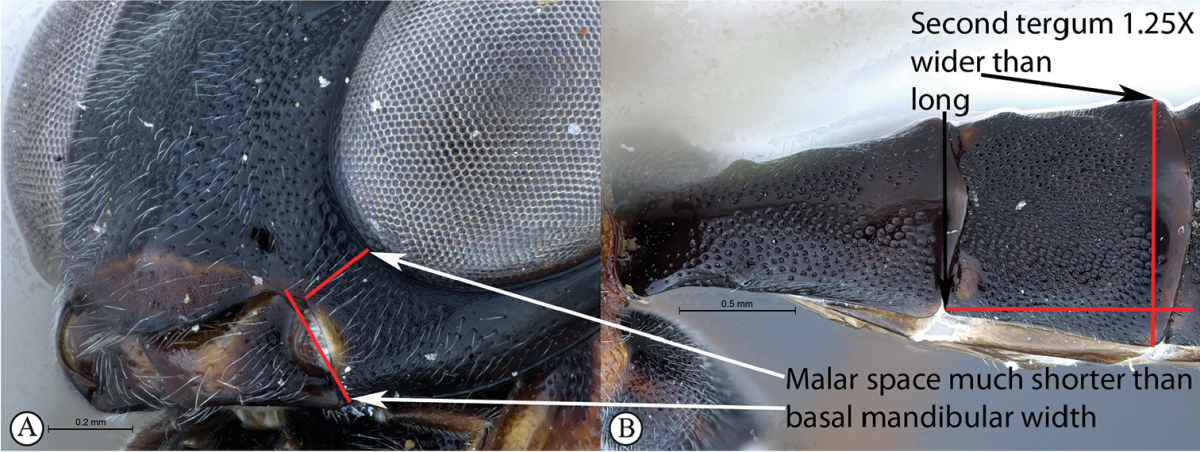	
9	Malar space 0.6 times as long as basal mandibular width (A). Second tergum posteriorly 1.25 times broader than long (B)	***Cryptopimpla zwarti* sp. n.**
	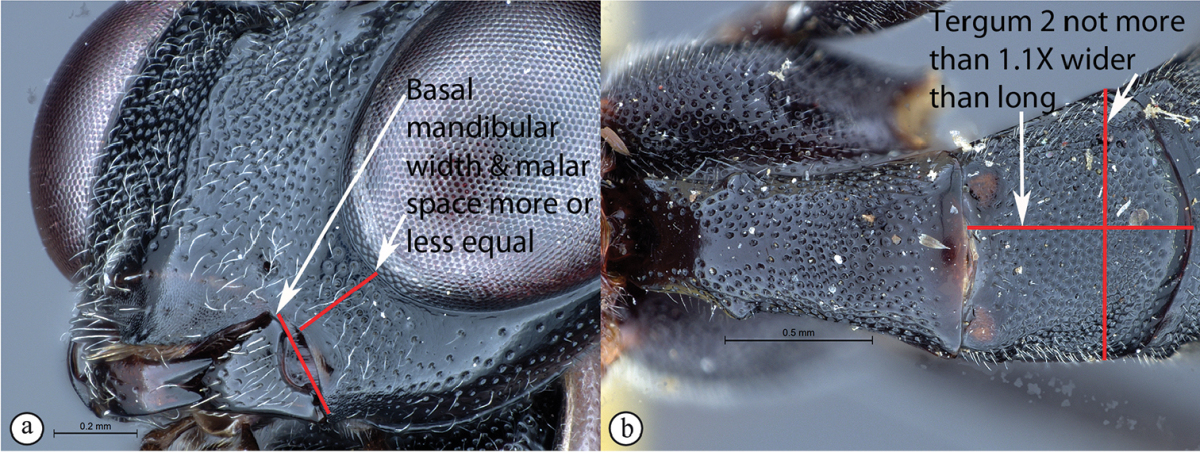	
–	Malar space 0.9–1.3 times as long as basal mandibular width (a). Tergum 2 posteriorly no more than 1.1 times broader than long (b)	***Cryptopimpla rubrithorax* Morley**

#### Cryptopimpla elongatus

Taxon classificationAnimaliaHymenopteraIchneumonidae

Reynolds Berry & van Noort sp. n.

http://zoobank.org/3BB8B8E3-343B-4CF3-BE7B-6E42D4AC1CE2

Fig. 1

##### Type material.

**HOLOTYPE** ♀: South Africa, Northern Cape, Hantam National Botanical Garden, 31°24.274'S, 19°09.164'E, 755m, 22 May–12 June 2008, S. van Noort, GL07-DOL1-M39, Malaise trap, Nieuwoudtville-Roggeveld Dolerite Renosterveld, SAM-HYM-P047468 (SAMC).

##### Description.

Body subpolished. Colour. Head black, clypeus and mouthparts dark brown. Body mostly rufescent apart from the mesonotum, black at the wing bases, mesopleuron ventrally black, submetapleural lobe black, fore and mid coxae black (remaining parts of front leg missing), trochanters and trochantellus of mid and hind legs black and terga 5–8 black.

Head. Densely punctate. Frons unarmed. Clypeus profile weakly convex with a curved lip on the ventral margin. Clypeus edge convex. Upper tooth of mandible longer than the lower tooth. Setae on head and clypeus short and sparse. Eye large and bulbous, maximum width in anterior view 0.73 times shortest inter-ocular distance, maximum width in lateral view 0.79 times maximum length. Tentorial pits small and indistinct. Flagellum tapered to a slender apex.

Mesosoma. Mesoscutum moderately punctate. Scuto-scutellar groove broad, with deep lateral indentations. Epicnemial carinae present ventrally and dorsally, dorsally converging toward anterior edge of mesopleuron. Propodeum without carinae, its anterior margin with a blunt median projection. Mid coxa posteriorly glossy and smooth. Wings hyaline, base of stigma brown. Fore wing with two bullae close together appearing as one; vein 2m-cu sinuate; areolet anteriorly truncate-shaped. Hind wing with one basal hamulus and six distal hamuli.

Metasoma. Tergum 1 longer than the hind coxae, terga 2 and 3 longer than wide; tergum 1 with dorsolateral carinae substituted by longitudinal wrinkles, densely punctate and with posterior margin weakly convex; second tergum 1.26 times longer than wide posteriorly, spiracle situated at basal 0.36 of tergum (measured in lateral view), gastrocoeli moderately large and circular; terga 4–8 slightly compressed; tergum 6 half as wide as tergum 5. Hypopygium strongly sclerotized. Ovipositor upcurved; sheath striations present.

CT 2.1; ML 0.9; IO 1.9; OO 1.7; Fl_1_ 5; OT 0.5; body length 6.7 mm; antenna length 8.4 mm; fore wing length 7.0 mm.

**Figure 1.**
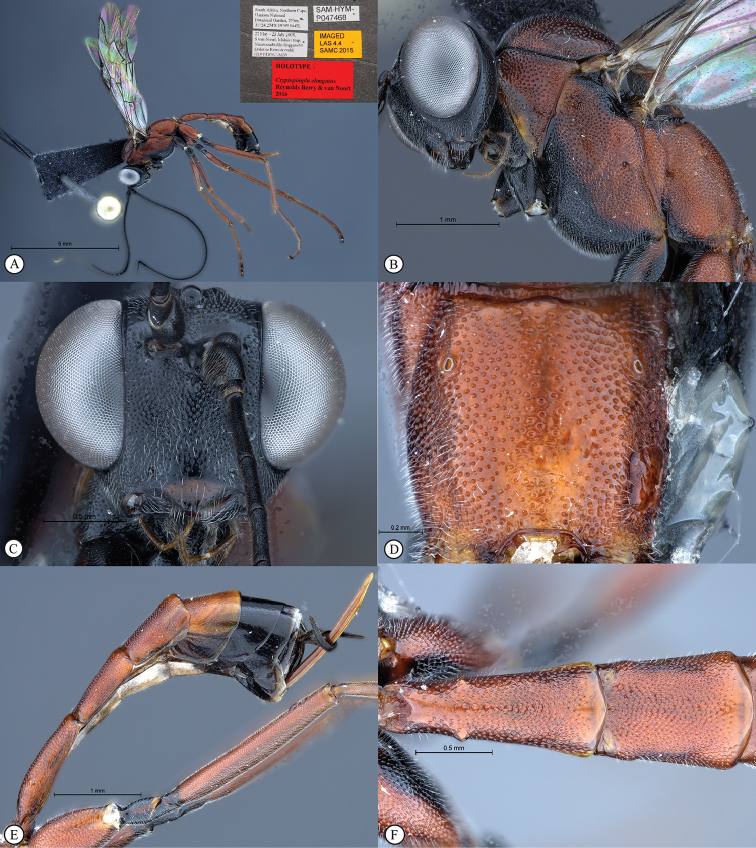
*Cryptopimpla elongatus*. Holotype. **A** Habitus, lateral view inset: data labels **B** Head and mesosoma, lateral view **C** Head, anterior view **D** Propodeum, dorsal view **E** Metasoma, lateral view **F** Metasomal terga 1 and 2, dorsal view.

##### Differential diagnosis.

*Cryptopimpla elongatus* can be distinguished from all other Afrotropical *Cryptopimpla* by having a more elongated metasoma, where terga 1–3 are longer than wide with the spiracle on tergum 2 situated at basal 0.36 of tergum (measured in lateral view). There are a few species with a rufescent/black colour combination, but *Cryptopimpla elongatus* is the only species to have rufescent legs (trochanter and trochantellus black) and a mostly rufescent metasoma (terga 5–8 black). The eye in anterior view is large, its maximum length 0.73 times the shortest inter-ocular distance, separating the species from all other Afrotropical *Cryptopimpla* whose eye length in anterior view is 0.38–0.66 times the shortest inter-ocular distance. A broad scuto-scutellar groove with deep lateral indentations distinguishes *Cryptopimpla elongatus* from closely-related species *Cryptopimpla fernkloofensis*, *Cryptopimpla hantami*, *Cryptopimpla neili*, and *Cryptopimpla parslactis*. The metasomal tergum 1 with dorsolateral carinae substituted with longitudinal wrinkles distinguishes *Cryptopimpla elongatus* from closely-related species *Cryptopimpla fernkloofensis* and *Cryptopimpla neili*. Gastrocoeli on tergum 2 are moderately large and circular, separating *Cryptopimpla elongatus* (and *Cryptopimpla fernkloofensis*) from the remaining closely-related species in the *rubrithorax* species-group.

##### Etymology.

The name refers to the rather elongated metasoma of this species. Noun in apposition.

##### Distribution.

South Africa (Northern Cape).

##### Comments.

This is a rare species known only from one female specimen collected in Nieuwoudtville-Roggeveld Dolerite Renosterveld. Intensive sampling in other areas of the Cape Floral Kingdom has produced no further specimens. The female metasoma is depressed (not slightly compressed) distinguishing *Cryptopimpla elongatus* from the closely-related species *Cryptopimpla onyxi*, *Cryptopimpla zwarti*, *Cryptopimpla hantami*, and *Cryptopimpla rubrithorax*. However, no female specimens are available for closely-related species *Cryptopimpla fernkloofensis*, *Cryptopimpla parslactis*, and *Cryptopimpla neili*, so no comparisons could be made with these species.

#### Cryptopimpla fernkloofensis

Taxon classificationAnimaliaHymenopteraIchneumonidae

Reynolds Berry & van Noort sp. n.

http://zoobank.org/EA5022CA-599E-4EA0-B032-0FF1EA108A08

Fig. 2

##### Type material.

**HOLOTYPE** ♂: South Africa, Western Cape, Fernkloof Nature Reserve, 33°39.941'S, 21°53.505'E, 300–340m, 13 May 1995, S. van Noort, Sweep, Mesic Mountain Fynbos, SAM-HYM-P008237 (SAMC).

##### Description.

Body subpolished. Colour. Body mostly fulvous, mesosoma ventrally black. Head black. Clypeus fulvous. Mandibles fulvous to black at apex. Mesoscutum and mesonotum dorsolaterally black. Submetapleural lobe black with various dark markings on legs. Metasomal terga 6–8 and male genitalia brown.

Head. Densely punctate. Frons unarmed. Setae on head and clypeus short and sparse. Clypeus profile weakly convex with a curved lip on the ventral margin. Clypeus edge convex. Flagellum tapered to a slender apex. Tentorial pits small and indistinct. Eye in lateral view 0.67 times as wide as long, moderately sized in anterior view with maximum width 0.54 times the shortest inter-ocular distance. Upper tooth of mandible longer than the lower tooth.

Mesosoma. Mesoscutum moderately punctate. Scutellar and metanotal margins tapered towards medial area. Epicnemial carinae present ventrally and dorsally, dorsally converging toward anterior edge of mesopleuron. Propodeum with carination reduced to medial area, its anterior margin with medial tooth. Wings hyaline. Fore wing with two bullae closely situated appearing as one; vein 2m-cu sinuate; areolet anteriorly truncate-shaped. Hind wing with one basal hamulus and six (left wing) to seven (right wing) distal hamuli.

Metasoma. Depressed; tergum 1 with a single carina ventrad of spiracle, densely punctate with posterior margin weakly convex; tergum 2 as long as it is broad posteriorly, spiracle situated at basal 0.24 of tergum (measured in lateral view), gastrocoeli moderately large and circular.

CT 2.0; ML 0.9; IO 1.7; OO 1.4; Fl_1_ 3.5; body length 9.4 mm; antenna length 9.9 mm; fore wing length 7.2 mm.

**Figure 2.**
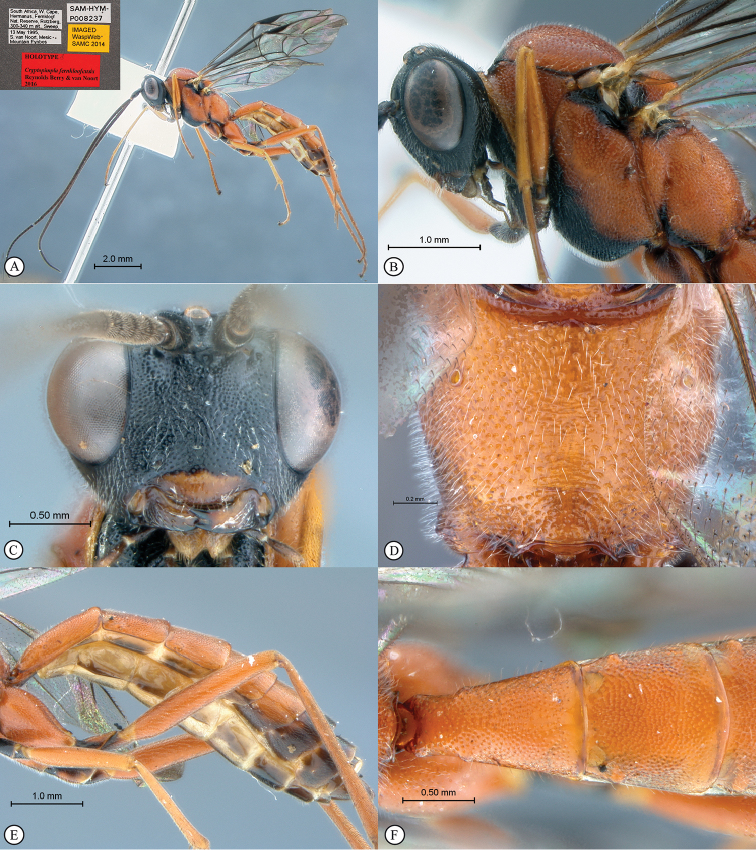
*Cryptopimpla fernkloofensis*. Holotype **A** Habitus, lateral view inset: data labels **B** Head and mesosoma, lateral view **C** Head, anterior view **D** Propodeum, dorsal view **E** Metasoma, lateral view **F** Metasomal terga 1 and 2, dorsal view.

##### Differential diagnosis.

*Cryptopimpla fernkloofensis* can be distinguished from all other Afrotropical *Cryptopimpla* species by having a mostly fulvous body with the mesosoma black ventrally, the head is black and the clypeus and mouthparts are fulvous in colour; the species is the largest of the *Cryptopimpla* species in the Afrotropical region with a body length of 9.4 mm, compared to other species that have body sizes less than 8.7 mm; the anterior propodeal margin of the species has a medial tooth; the scuto-scutellar groove is narrow, without deep lateral indentations; and the scutellar and metanotal margins distinctly taper towards the medial area. The gastrocoeli on the metasomal tergum 2 are moderately large and circular, which distinguishes *Cryptopimpla fernkloofensis* (and *Cryptopimpla elongatus*) from all other closely-related species in the *rubrithorax* species-group. The presence of a single carina ventrad of the spiracle on the metasomal tergum 1, without wrinkles, separates *Cryptopimpla fernkloofensis* from closely-related species in the *rubrithorax* species-group.

##### Etymology.

Named after the type locality. Noun in apposition.

##### Distribution.

South Africa (Western Cape).

##### Comments.

This is a rare species known only from one male specimen collected in Mesic Mountain Fynbos. Intensive sampling in other areas of the Cape Floral Kingdom, including Mesic Mountain Fynbos at various other localities, has produced no further specimens.

#### Cryptopimpla goci

Taxon classificationAnimaliaHymenopteraIchneumonidae

Reynolds Berry & van Noort sp. n.

http://zoobank.org/DA40C867-142C-483B-8F3C-E3F55BA3BCC0

Fig. 3

##### Type material.

**HOLOTYPE** ♂: South Africa, Western Cape, Koeberg Nature Reserve, 33°37.622'S, 18°24.259'E, 741m, 3 - 31 October 1997, S. van Noort, KO97-M12, Malaise trap, West Coast Strandveld, SAM-HYM-P0474345 (SAMC).

##### Description.

Body subpolished. Colour. Head white with a median black band on the face, frons, reaching around ocelli and occiput black, not reaching the eyes. Body mostly fulvous, mesoscutum black anteromedially, extending about 0.8 length of mesoscutum.

Head. Finely punctate. Frons unarmed. Setae on head and clypeus short and sparse. Flagellum tapered to a slender apex. Clypeus profile convex, bulbous. Clypeus edge convex. Upper tooth of mandible longer than the lower tooth. Tentorial pits large and distinct. Eye in lateral view 0.69 times as long as wide, narrow in anterior view with maximum width 0.4 times shortest inter-ocular distance.

Mesosoma. Mesosocutum moderately punctate. Scuto-scutellar groove broad. Epicnemial carinae present ventrally and dorsally, dorsally converging toward anterior edge of mesopleuron. Propodeum with anterior margin medially straight, carination include pleural carinae and a well-defined posterior transverse carina. Wings hyaline; fore wing with two bullae closely situated appearing as one; vein 2m-cu sinuate; areolet anteriorly truncate-shaped. Hind wing with one basal hamulus and seven distal hamuli.

Metasoma. Depressed; tergum 1 impunctate with distinct dorsolateral carinae present as a carina ventrad of spiracle, with a secondary carina leading from the ventral carina to the spiracle, posterior margin weakly convex; second tergum 1.21 times longer than broad, spiracle situated at basal 0.35 of tergum (measured in lateral view), gastrocoeli elongate.

CT 1.8; ML 0.75; IO 1.6; OO 1.1; Fl_1_ 3.9; body length 7.5 mm; antenna length 7.2 mm; fore wing length 5.3 mm.

**Figure 3.**
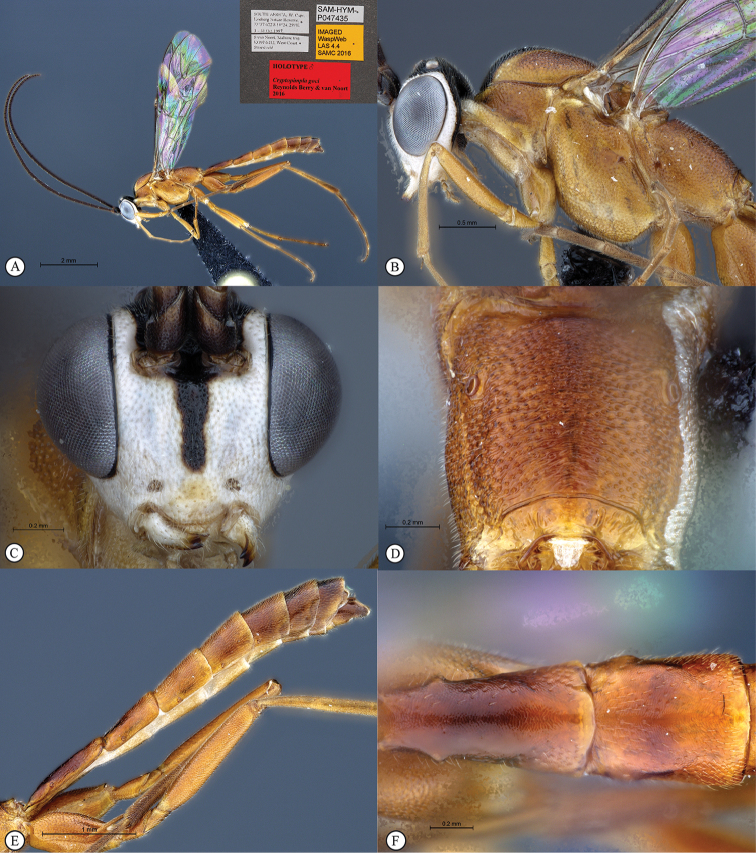
*Cryptopimpla goci*. Holotype **A** Habitus, lateral view inset: data labels **B** Head and mesosoma, lateral view **C** Head, anterior view **D** Propodeum, dorsal view **E** Metasoma, lateral view **F** Metasomal terga 1 and 2, dorsal view.

##### Differential diagnosis.

*Cryptopimpla goci* is immediately distinguishable from all other Afrotropical *Cryptopimpla* species by having a colour combination of a largely fulvous body, with a white head. A short distinct carina leads to the spiracle from the base of a single carina ventrad of the spiracle which is unique in this species. *Cryptopimpla goci* is closely-related to *Cryptopimpla kogelbergensis* as both species (*goci* species-group) share a truly distinctive and well-defined posterior transverse carina, possess pleural carinae, a convex and bulbous clypeus without a curved lip on the ventral margin, and large tentorial pits distinguishing them from all other Afrotropical *Cryptopimpla* species in the *rubrithorax* species-group. The head is finely punctate, the maximum width of the eye in anterior view 0.38 times the shortest inter-ocular distance; the malar space 0.75 times the basal mandibular width; the length of the first flagellomere 3.9 times longer than wide; 2m-cu on the fore wing is sinuate; metasomal tergum 1 with a short distinct carina that leads to the spiracle from the base of a single carina ventrad of the spiracle; the second tergum 1.21 times longer than wide with elongate gastrocoeli separates *Cryptopimpla goci* from *Cryptopimpla kogelbergensis* where the head is smooth, the maximum width of the eye is much broader at 0.6–0.63 times the shortest inter-ocular distance; the malar space is 1.2 times the basal mandibular width; the length of the first flagellomere is 6.1–6.8 times longer than wide; vein 2m-cu on the fore wing is straight; dorsolateral carinae on the metasomal tergum 1 are substituted with longitudinal wrinkles and the second tergum is 1.0–1.04 times as long as wide with the gastrocoeli small and indistinct.

##### Etymology.

Named after the late Nosiphiwo Goci who worked as a research assistant in the Natural History Department of the Iziko South African Museum for over 17 years and whose immense contribution to the curation and digitization of the SAMC Hymenoptera collection warrants recognition. Noun in the genitive case.

##### Distribution.

South Africa (Western Cape).

##### Comments.

A rare species known only from one specimen collected in West Coast Strandveld in the Koeberg Nature Reserve as part of a continuous 13 month sampling inventory of the reserve using a variety of methods including Malaise traps, yellow pan traps and sweeping. Similar intensive sampling in other areas of the Cape Floral Kingdom, including sampling West Coast Strandveld at numerous other localities, produced no further specimens.

#### Cryptopimpla hantami

Taxon classificationAnimaliaHymenopteraIchneumonidae

Reynolds Berry & van Noort sp. n.

http://zoobank.org/68B27C7F-B8E8-4426-959D-FE58A7D6E89D

Fig. 4

##### Type material.

**HOLOTYPE** ♀: South Africa, Northern Cape, Hantam National Botanical Garden, 31°24.182'S, 19°08.587'E, 741m, 17 March - 21 April 2008, S. van Noort, GL07-REN3-M24, Malaise trap, Nieuwoudtville Shale Renosterveld, SAM-HYM-P047467 (SAMC). **PARATYPE** ♂: South Africa, Northern Cape, Hantam National Botanical Garden, 31°24.182'S, 19°08.587'E, 741m, 21 April – 22 May 2008, S. van Noort, GL07-REN3-M31, Malaise trap, Nieuwoudtville Shale Renosterveld, SAM-HYM-P047469 (SAMC).

##### Description.

Body moderately covered in short setae. Colour. Head black, clypeus testaceous; mesosoma dark fulvous; pronotal collar and anterior corner of mesopleuron slightly lighter, propleuron ventrally black, metanotum black at the wing bases, sternum of mesothorax with small black spot; metasoma black, terga 6–8 white at the posterior margins; middle and hind legs black to dark brown with medial dark fulvous longitudinal bands on the coxae, front leg black to testaceous toward apex.

Head. Matt. Frons unarmed. Clypeus profile weakly convex with a curved lip on the ventral margin. Clypeus edge convex. Upper tooth of mandible longer than the lower tooth. Head densely punctate. Eye in lateral view 0.73 times as broad as long, maximum width in anterior view half the shortest inter-ocular distance. Tentorial pits small and indistinct. Malar space 0.8–1.0 times basal mandibular width. Flagellum tapered to a slender apex.

Mesosoma. Matt. Mesosocutum moderately punctate. Scuto-scutellar excavation shallow. Epicnemial carinae present ventrally and dorsally, dorsally converging toward anterior edge of mesopleuron. Propodeum without carinae, its anterior margin with a blunt median projection. Wings hyaline. Fore wing with two bullae close together appearing as one; vein 2m-cu sinuate; areolet anteriorly truncate-shaped. Hind wing with one basal hamulus and six distal hamuli.

Metasoma. Subpolished. Terga 4–8 slightly compressed in female; tergum 1 with dorsolateral carinae substituted with longitudinal wrinkles, densely punctate, posterior margin weakly convex; second tergum 1.06–1.32 times longer than wide, spiracle situated at basal 0.27 of tergum (measured in lateral view), gastrocoeli small and indistinct; tergum 6 half as wide as tergum 5; hypopygium in female strongly sclerotized. Ovipositor upcurved; sheath striations present.

CT 2–2.1; ML 0.8–1; IO 2.3–2.4; OO 1.8–2.1; OT 0.5 (single female); Fl_1_ 3.7–5.6; body length 8.1–8.9 mm; antenna length 7.9 mm (males antennae intact); fore wing length 5.8–6.2 mm.

Male: Propleuron and pronotum completely black, clypeus testaceous rather than dark brown; colouration of the legs as in female except fulvous bands on mid and hind coxae are lacking and terga 6–8 not white at the posterior margins. Males are more setose; metasoma depressed, tergum 6 half as wide as 5.

**Figure 4.**
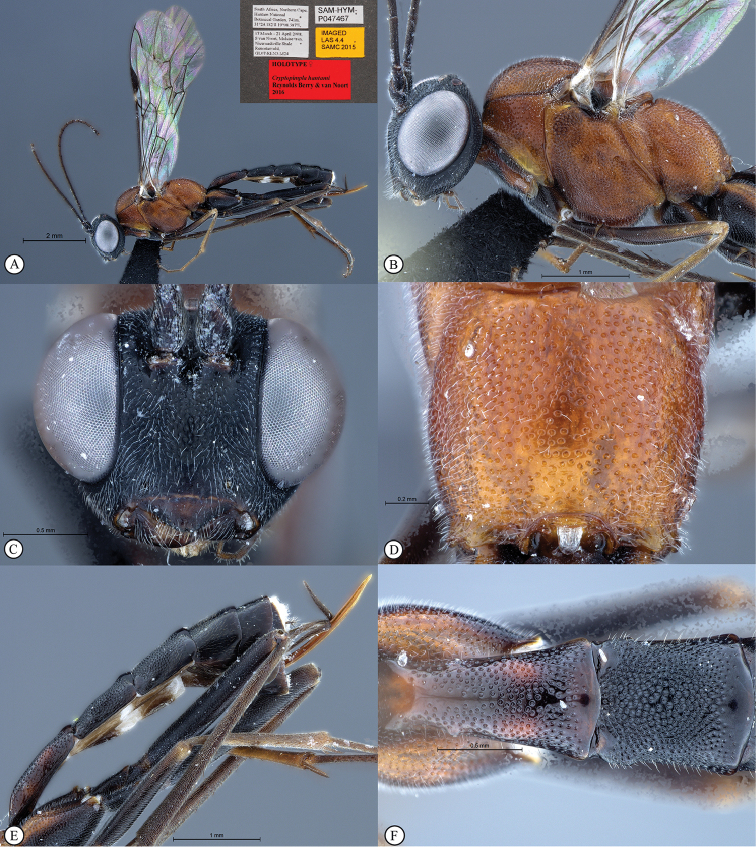
*Cryptopimpla hantami*. Holotype **A** Habitus, lateral view inset: data labels **B** Head and mesosoma, lateral view **C** Head, anterior view **D** Propodeum, dorsal view **E** Metasoma, lateral view **F** Metasomal terga 1 and 2, dorsal view.

##### Differential diagnosis.

*Cryptopimpla hantami* is distinguishable from all species in the *rubrithorax* species-group by having a matt head and mesosoma, with the body moderately covered in short setae, rather than possessing a subpolished body sparsely covered in setae. The presence of a shallow scuto-scutellar excavation and small and indistinct gastrocoeli on the metasomal tergum 2 distinguishes *Cryptopimpla hantami* (and *Cryptopimpla neili*) from other species in the *rubrithorax* species-group where a broad groove with or without deep lateral indentations may be present and the gastrocoeli are moderately large and distinct. The metasomal tergum 1 with dorsolateral carinae substituted with longitudinal wrinkles distinguishes *Cryptopimpla hantami* from closely-related species *Cryptopimpla fernkloofensis* and *Cryptopimpla neili*.

##### Distribution.

South Africa (Northern Cape).

##### Etymology.

Named after the type locality. Hantam National Botanical Garden. Noun in apposition.

#### Cryptopimpla kogelbergensis

Taxon classificationAnimaliaHymenopteraIchneumonidae

Reynolds Berry & van Noort sp. n.

http://zoobank.org/38A172D2-39B6-45E6-8C58-0DF55F06EAA9

Fig. 5

##### Material examined.

**HOLOTYPE** ♀: South Africa, Western Cape, Kogelberg Nature Reserve, 34°16.481'S, 19°01.033'E, 118m, 16 May – 16 June 1999, S. van Noort, KO98-M23, Malaise trap, Mesic Mountain Fynbos, last burnt c. 1988, SAM-HYM-P047475 (SAMC). **Paratypes**: ♀: South Africa, Western Cape, Gamkaberg Nature Reserve, 33°39.504'S, 21°54.947'E, 322m, 30 March 2010 - 24 July 2010, S. van Noort, GB09-SUC04-M38, Malaise trap, Gamka thicket, SAM-HYM-P044551 (SAMC). 2♀: South Africa, Northern Cape, Hantam National Botanical Garden, 31°24.182'S, 19°08.587'E, 22 May – 23 July 2008, S. van Noort, GL07-REN3-M38, 741m, Malaise trap, Nieuwoudtville Shale Renosterveld, SAM-HYM-P047463 (SAMC, BMNH). ♂ South Africa, Western Cape, Gamkaberg Nature Reserve, 33°43.745'S, 21°56.922'E, 1000m, 10 Sept – 4 Nov 2009, S. van Noort, GB09-REN1-Y38, Yellow pan trap, Renosterveld, SAM-HYM-P061546 (SAMC).

##### Description.

Body subpolished. Colour. Head white with a median brown band on the face and brown spots at tentorial pits. Mesoscutum brown dorsolaterally and anteromedially; medially fulvous basad of black colouration; grey or cream stripes medially, extending about 0.8 length of mesoscutum. Mesosoma colour combination brown, white and black; dorsally mostly brown, ventrally black, laterally white. Scutellum and mesonotum black dorsolaterally, testaceous medially. Propodeum mostly brown to yellowish testaceous, black anteriorly. Legs, antennae and metasoma yellowish testaceous with variable dark markings on terga.

Head. Smooth, impunctate. Frons unarmed. Setae on head and clypeus short and sparse. Flagellum tapered to a slender apex. Clypeus profile distinctly convex and bulbous. Clypeus edge convex. Upper tooth of mandible longer than the lower tooth. Tentorial pits large and distinct. Eye in lateral view 0.74–0.76 times as broad as long, maximum width in anterior view 0.6–0.63 times shortest inter-ocular distance.

Mesosoma. Mesosocutum moderately punctate. Scuto-scutellar groove broad. Epicnemial carinae present ventrally and dorsally, dorsally converging toward anterior edge of mesopleuron. Propodeum with anterior margin medially straight, but may have a blunt medial projection; carination include pleural carinae and a well-defined posterior transverse carina. Wings hyaline. Fore wing with two bullae closely situated appearing as one or separated; vein 2m-cu straight; areolet anteriorly truncate-shaped. Hind wing with one or two basal hamuli and six to seven distal hamuli.

Metasoma. Depressed; tergum 1 with dorsolateral carinae substituted with longitudinal wrinkles, impunctate, posterior margin weakly convex; second tergum 0.96–1 times as long as wide, spiracle situated at basal 0.21–0.23 of tergum (measured in lateral view), gastrocoeli small to indistinct; tergum 6 half as wide as tergum 5; hypopygium moderately sclerotized. Ovipositor upcurved; sheath striations present.

CT 1.9–2.0; ML 1.2; IO 2.0; OO 1.3; Fl_1_ 5.1–6.1; OT 0.5–0.7; body length 4.2–5.6 mm; antenna length 6.3–6.5 mm; fore wing length 4.6–5.1 mm.

**Figure 5.**
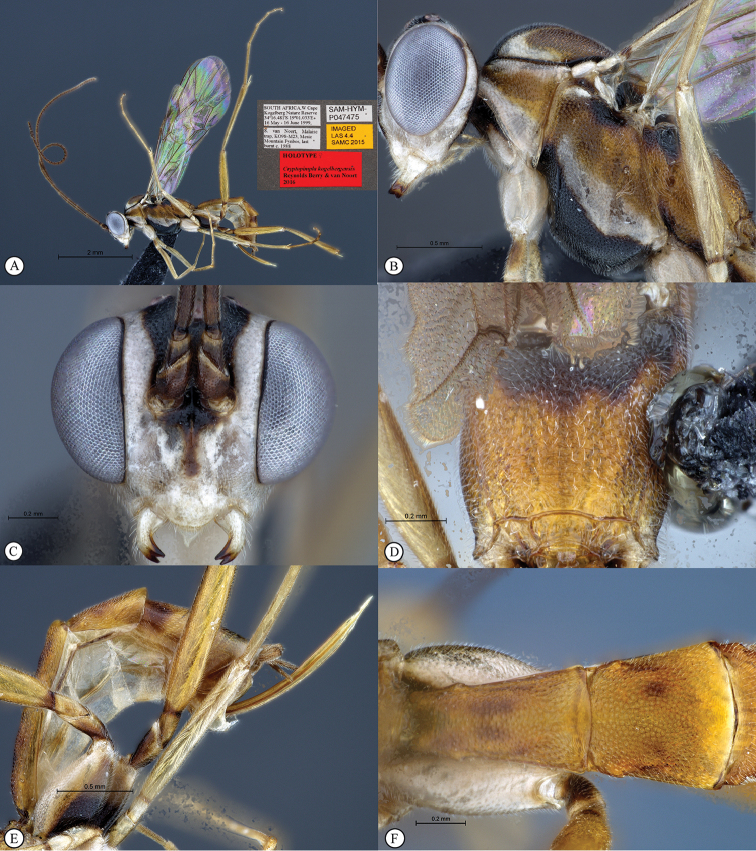
*Cryptopimpla kogelbergensis*. Holotype **A** Habitus, lateral view **B** Head and mesosoma, lateral view **C** Head, anterior view **D** Propodeum, dorsal view **E** Wings inset: data labels **F** Metasoma, lateral view.

##### Differential diagnosis.

*Cryptopimpla kogelbergensis* is immediately distinguishable from all other Afrotropical *Cryptopimpla* species by the distinctive colour combination of the mesopleuron, which is three-banded in brown, white and black; possession of a smooth head; and vein 2m-cu is straight on the fore wing. The maximum width of the eye in anterior view 0.6–0.63 times the shortest inter-ocular distance; the length of the first flagellomere 6.1–6.8 times longer than wide; the metasomal tergum 1 with dorsolateral carinae substituted with longitudinal wrinkles; and the metasomal tergum 2 as long as wide with small and distinct gastrocoeli distinguishes *Cryptopimpla kogelbergensis* from *Cryptopimpla goci* where the maximum width of the eye in the anterior view is much narrower at 0.38 times the shortest inter-ocular distance; distinct dorsolateral carinae are presented as a short carina leading from a single carina ventrad of the spiracle; the length of the first flagellomere is 3.9 times longer than wide; vein 2m-cu on the fore wing is sinuate; and the second tergum is 1.21 times longer than wide with the gastrocoeli elongate.

##### Etymology.

Named after the type locality. Noun in apposition.

##### Distribution.

South Africa (Western Cape & Northern Cape).

#### Cryptopimpla neili

Taxon classificationAnimaliaHymenopteraIchneumonidae

Reynolds Berry & van Noort sp. n.

http://zoobank.org/E1A7879E-E44A-46E1-8CF2-EC7BD080DCB4

Fig. 6

##### Material examined.

**HOLOTYPE** ♂: South Africa, Western Cape, Kogelberg Nature Reserve, 34°16.481'S, 19°01.033'E, 118m, 16 March 1999 - 16 April 1999, S. van Noort, KO98-M18, Malaise trap, Mesic Mountain Fynbos, last burnt c. 1988, SAM-HYM-P047436 (SAMC).

##### Description.

Body subpolished. Colour. Head black, clypeus and mandibles white to brown; white markings on either side of toruli. Body mostly fulvous with dark markings on metanotum and metasomal terga 5–8, pronotal collar white.

Head. Densely punctate. Frons unarmed. Setae on head and clypeus short and sparse. Eye in lateral view 0.74 times as wide as long. Shortest inter-ocular distance 1.94 times maximum eye width in anterior view. Flagellum tapered to a slender apex. Clypeus profile weakly convex with a curved lip on the ventral margin. Clypeus edge convex. Upper tooth of mandible longer than the lower tooth. Tentorial pits small or indistinct.

Mesosoma. Mesosocutum moderately punctate. Shallow excavation separates mesoscutum from scutellum. Epicnemial carinae present ventrally and dorsally, dorsally converging toward anterior edge of mesopleuron. Anterior propodeal margin with a blunt median projection; carination absent. Wings hyaline. Fore wing with two bullae closely situated appearing as one; vein 2m-cu sinuate; areolet anteriorly truncate-shaped. Hind wing with two basal hamuli and six distal hamuli.

Metasoma. Tergum 1 densely punctate, lacking dorsolateral carinae, posterior margin medially tapered to a point; second tergum 1.07 times longer than broad, spiracle situated at basal 0.28 of tergum (measured in lateral view), gastrocoeli small and elliptic; terga 4–8 strongly compressed.

CT 2.0; ML 1.0; IO 2.3; OO 1.6; Fl_1_ 5.0; body length 7.5 mm; antenna length 8.5 mm; fore wing 6.9 mm.

**Figure 6.**
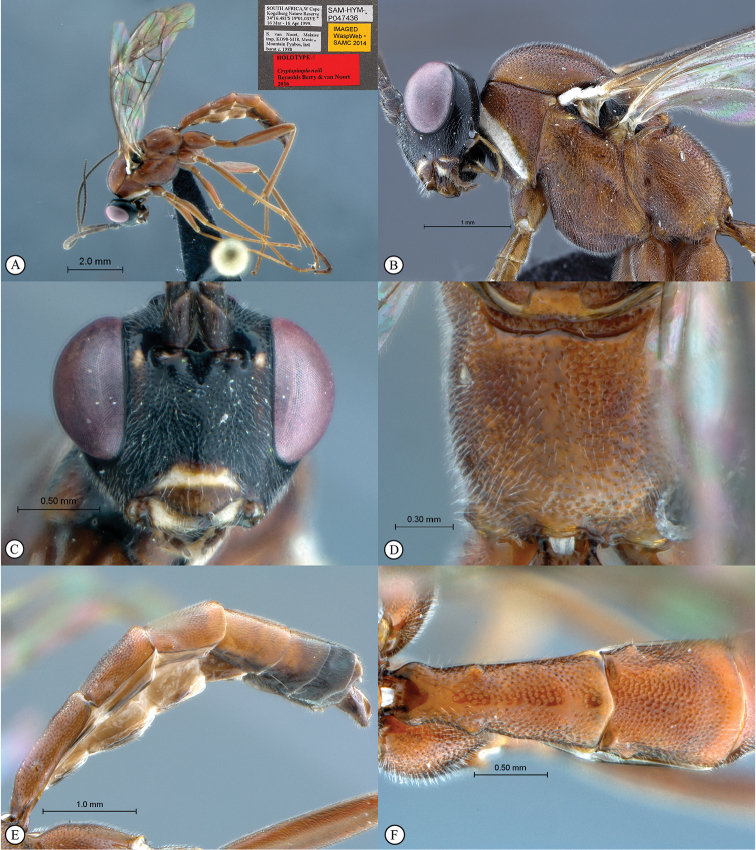
*Cryptopimpla neili*. Holotype **A** Habitus, lateral view inset: data labels **B** Head and mesosoma, lateral view **C** Head, anterior view **D** Propodeum, dorsal view **E** Metasoma, lateral view **F** Metasomal terga 1 and 2, dorsal view.

##### Differential diagnosis.

*Cryptopimpla neili* is closely-related to *Cryptopimpla hantami* because both species exclusively possess a shallow excavation separating the mesosocutum from the scutellum and the presence of small elliptic gastrocoeli on the second tergum, whereas a groove is present and the shape of the gastrocoeli is large or elongate in the other species in the *rubrithorax* species-group. *Cryptopimpla neili* is immediately distinguishable from all other Afrotropical *Cryptopimpla* species, including *Cryptopimpla hantami*, by having a unique colour combination of a fulvous body, white pronotal collar and a clypeus distinguished by two colours; and the metasomal tergum 1 lacking dorsolateral carinae with the posterior margin medially tapered to a point.

##### Etymology.

Named after the first author's father. Noun in the genitive case.

##### Distribution.

South Africa (Western Cape).

##### Comments.

A rare species known only from one specimen. Intensive sampling in other areas of the Cape Floral Kingdom produced no further specimens. The metasomal terga 4–8 of the male are strongly compressed and this separates the species from the closely-related species *Cryptopimpla fernkloofensis*, *Cryptopimpla hantami*, *Cryptopimpla parslactis*, *Cryptopimpla rubrithorax*, and *Cryptopimpla onyxi*. However, no male specimens are available for the remaining species *Cryptopimpla elongatus* and *Cryptopimpla zwarti* within the *rubrithorax* species-group. Thus, no comparisons could be made with those species.

#### Cryptopimpla onyxi

Taxon classificationAnimaliaHymenopteraIchneumonidae

Reynolds Berry & van Noort sp. n.

http://zoobank.org/E43A6387-8EF2-4629-B688-A58EF7096B45

Fig. 7

##### Material examined.

**HOLOTYPE** ♀: South Africa, Western Cape, Walker Bay Nature Reserve, 34°27.414'S, 19°21.393'E, 57m, 14 May –14 June 1997, S. van Noort, WB97-M01, Malaise trap, South coast Strandveld, SAM-HYM-P047460 (SAMC). **Paratypes** 7♂: South Africa, Western Cape, Walker Bay Nature Reserve, 34°27.414'S, 19°21.393'E, 57m, 6 September – 4 October 1997, S. van Noort, WB97-M09, Malaise trap, South coast Strandveld, SAM-HYM-P044545, SAM-HYM-P047478, SAM-HYM-P047479, SAM-HYM-P047481 (SAMC, BMNH); ♂: South Africa, Western Cape, Walker Bay Nature Reserve, 34°27.414'S, 19°21.393'E, 57m, 18 Apr – 16 May 1998, S. van Noort, WB97-M30, Malaise trap, South coast Strandveld, SAM-HYM-P048105 (SAMC); ♂: South Africa, Western Cape, Kogelberg Nature Reserve, 34°16.481'S, 19°01.033'E, 16 Mar – 16 Apr 1999, S. van Noort, KO98-M17, Malaise trap, Mesic Mountain Fynbos last burnt c. 1988, SAM-HYM-P47482 (SAMC).

##### Description.

Body subpolished. Body black. Pronotal collar white.

Head. Densely punctate. Frons unarmed. Clypeus profile weakly convex with a curved lip on the ventral margin. Clypeus edge convex. Upper tooth of mandible longer than the lower tooth. Setae on head and clypeus short and sparse. Flagellum tapered to a slender apex. Tentorial pits small or indistinct. Eye in lateral view 0.7–0.72 times as long as wide, maximum width in anterior view 0.4–0.56 times shortest inter-ocular distance.

Mesosoma. Mesosocutum moderately punctate. Scuto-scutellar groove broad with deep lateral indentations. Epicnemial carinae present ventrally and dorsally, dorsally converging toward anterior edge of mesopleuron. Propodeum with carination reduced to medial area or absent, its anterior margin with a blunt median projection. Wings hyaline. Fore wing with two bullae closely situated appearing as one; vein 2m-cu sinuate; areolet truncate-shaped. Hind wing with one or two basal hamuli and six to seven distal hamuli.

Metasoma. Tergum 1 punctate with dorsolateral carinae substituted with longitudinal wrinkles, posterior margin weakly convex; second tergum 1.09–1.25 times longer than wide, spiracle situated at basal 0.25–0.26 of tergum (measured in lateral view), gastrocoeli elongate; terga 4–8 moderately compressed in females, no dorsolateral compression in males; female metasomal tergum 5 as wide as tergum 6; hypopygium strongly sclerotized. Ovipositor weakly upcurved; sheath striations present.

CT 1.6–1.8; ML 0.9–0.96; IO 2.1; OO 1.9; Fl_1_ 4.2; OT 0.5 (SAM-HYM-P047460); body length 6–8.4 mm; antenna length 8.1–9.0 mm; fore wing length 6.1–6.8 mm.

**Figure 7.**
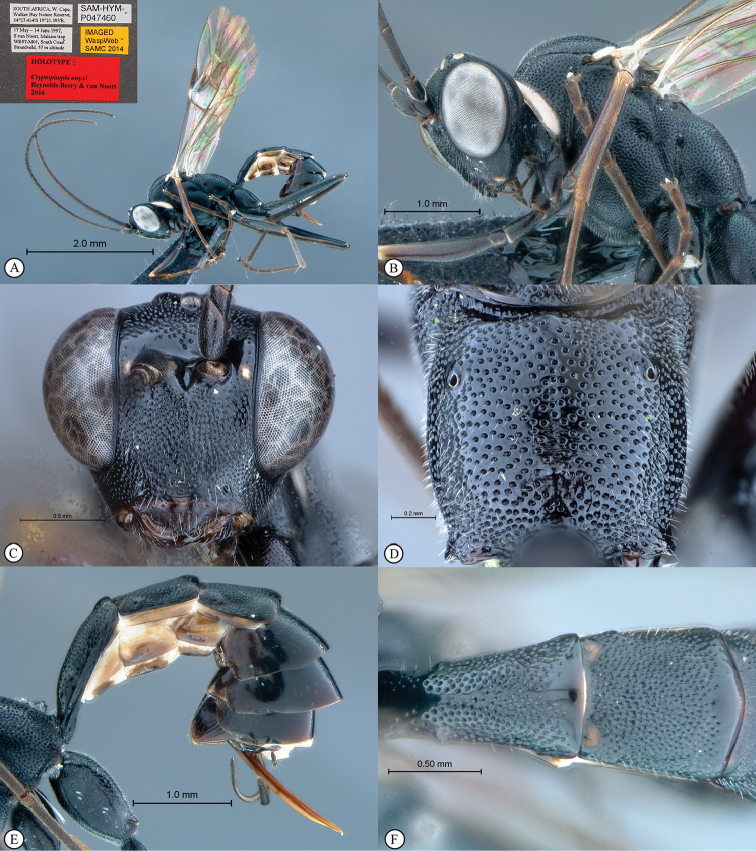
*Cryptopimpla onyxi*. Holotype **A** Habitus, lateral view inset: data labels **B** Head and mesosoma, lateral view **C** Head, anterior view **D** Propodeum, dorsal view **E** Metasoma, lateral view **F** Metasomal terga 1 and 2, dorsal view.

##### Differential diagnosis.

*Cryptopimpla onyxi* is immediately distinguishable from all other Afrotropical *Cryptopimpla* species by having a unique colour combination of a black body and a white pronotal collar. The clypeus is 1.6–1.8 times broader than high, distinguishing *Cryptopimpla onyxi* from all other species in the *rubrithorax* species-group where the clypeus is more than 1.8 times broader than high. The scuto-scutellar groove in *Cryptopimpla onyxi* is broad with deep lateral indentations, distinguishing the species from closely-related species *Cryptopimpla fernkloofensis*, *Cryptopimpla parslactis*, *Cryptopimpla hantami*, and *Cryptopimpla neili*. The metasomal tergum 1 with dorsolateral carinae substituted with longitudinal wrinkles distinguishes the species from *Cryptopimpla fernkloofensis* and *Cryptopimpla neili*. Gastrocoeli on tergum 2 are elongate, separating *Cryptopimpla onyxi* from closely-related species *Cryptopimpla fernkloofensis*, *Cryptopimpla elongatus*, *Cryptopimpla neili*, and *Cryptopimpla hantami*.

**Etymology.** The species epithet refers to the black colouration of this species. Noun in apposition.

##### Distribution.

Occurs in Strandveld and Mountain Fynbos vegetation types in South Africa (Western Cape).

##### Comments.

The female metasomal tergum 5 as wide as tergum 6 separates the species from closely-related species *Cryptopimpla hantami*, *Cryptopimpla zwarti*, *Cryptopimpla elongatus*, and *Cryptopimpla rubrithorax* where tergum 5 is half as wide as high. However, no female specimens are available for *Cryptopimpla fernkloofensis*, *Cryptopimpla parslactis*, and *Cryptopimpla neili*. Thus, no comparisons could be made with those closely-related species.

#### Cryptopimpla parslactis

Taxon classificationAnimaliaHymenopteraIchneumonidae

Reynolds Berry & van Noort sp. n.

http://zoobank.org/8B2E756B-4869-4E1B-9AD5-89C52092D0D2

Fig. 8

##### Type material.

**HOLOTYPE** ♂: South Africa, Northern Cape, Hantam National Botanical Garden, 31°23.802'S, 19°08.799'E, 752m, 23 July–23 Aug 2008, S. van Noort, GL07-REN1-M43, Malaise trap, Nieuwoudtville Shale Renosterveld, SAM-HYM-P044547 (SAMC).

##### Description.

Body subpolished. Colour. Head and mesosoma mostly black, with the exception of the medial region of the mesopleuron and the propodeum that is orange. Legs with fore and mid coxae, trochanters and trochantellus black. Terga 2–8 mostly black, tergum 2 medially orange and terga 7–8 white posteriorly. Femora 1–2 black to light orange. Tibia and tarsus of front leg light orange. Tibia 2 light orange, tarsus 2 brown. Femora 3 orange, tibia and tarsus of hind leg brown.

Head. Densely punctate. Frons unarmed. Clypeus profile weakly convex with a curved lip on the ventral margin. Clypeus edge convex. Upper tooth of mandible longer than the lower tooth. Setae on head and clypeus short and sparse. Tentorial pits small and indistinct. Flagellum tapered to a slender apex. Eye in lateral view 0.7 times as long as wide, maximum width in anterior view 0.46 times shortest inter-ocular distance.

Mesosoma. Scuto-scutellar groove broad. Mesoscutum with fewer punctures inward of wing base. Epicnemial carinae present ventrally and dorsally, dorsally converging toward anterior edge of mesopleuron. Propodeum without carinae, its anterior margin with a weak and blunt medial projection. Wings slightly infuscate, venation dark. Fore wing with two bullae close together appearing as one; vein 2m-cu sinuate; areolet truncate-shaped. Hind wing with one basal hamulus and six distal hamuli.

Metasoma. Depressed. Tergum 1 with dorsolateral carinae substituted with longitudinal wrinkles, densely punctate, with posterior margin weakly convex; tergum 2 of metasoma 1.09 times as long as wide posteriorly, spiracle situated at basal 0.28 of tergum (measured in lateral view), gastrocoeli elongate; tergum 6 as wide as tergum 5.

CT 2.3; ML 0.92; IO 2.6; OO 2.0; body length 7.4 mm; fore wing length 7.0 mm.

**Figure 8.**
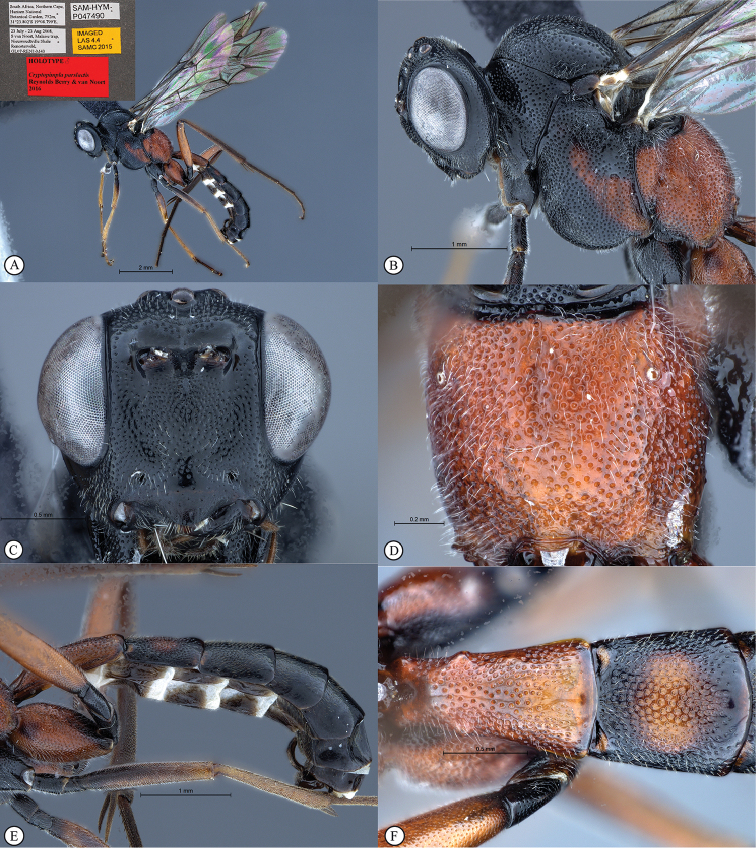
*Cryptopimpla parslactis*. Holotype **A** Habitus, lateral view inset: data labels **B** Head and mesosoma, lateral view **C** Head, anterior view **D** Propodeum, dorsal view **E** Metasoma, lateral view **F** Metasomal terga 1 and 2, dorsal view.

##### Differential diagnosis.

*Cryptopimpla parslactis* is immediately diagnosable from other Afrotropical *Cryptopimpla* by being the only species to have slightly infuscate wings with darker venation. *Cryptopimpla parslactis* is distinguishable from closely-related species in the *rubrithorax* species-group that have a rufous and black colour combination, by having a completely black mesoscutum and a combination of a mostly black metasoma with tergum 1 completely rufescent. In addition, while punctuation on the mesoscutum in the dorsal view is common amongst all the species, fewer punctures on the mesoscutum exist inward of the wing bases of *Cryptopimpla parslactis*. The metasomal tergum 1 with dorsolateral carinae substituted with longitudinal wrinkles distinguishes *Cryptopimpla parslactis* from closely-related species *Cryptopimpla fernkloofensis* and *Cryptopimpla neili*. Gastrocoeli on tergum 2 are elongate separating *Cryptopimpla parslactis* from closely-related species *Cryptopimpla fernkloofensis*, *Cryptopimpla elongatus*, and *Cryptopimpla hantami*.

##### Etymology.

So named because the wings are not quite hyaline, but rather slightly infuscate with a creamy-brown colour, “pars” meaning wing and “lactis” meaning cream. Noun in apposition.

##### Distribution.

South Africa (Northern Cape).

##### Comments.

A rare species known only from one specimen. Intensive sampling in other areas of the Cape region produced no further specimens.

#### Cryptopimpla rubrithorax

Taxon classificationAnimaliaHymenopteraIchneumonidae

Morley, 1916

Fig. 9

##### Material examined.

**HOLOTYPE** ♀: South Africa, Western Cape, Elsenberg, 11 October 1914, Mally and Petty, SAM-HYM-P000874 (SAMC). **Additional material**: ♀ South Africa, Western Cape, Kogelberg Nature Reserve, 34°16.481'S, 19°01.033"E, 16 September 1999, S. van Noort, KO98-M40, Malaise trap, Mesic mountain fynbos last burnt c. 1988, SAM-HYM-P044558 (SAMC). 2♀, 3♂ South Africa, Western Cape, Koeberg Nature Reserve, 33°37.622'S, 18°24.259'E, 8 August – 5 September 1997, S. van Noort, KO97-M07, KO97-M08, Malaise trap, West Coast Strandveld, SAM-HYM-P047461 (SAMC, BMNH). 1♀ South Africa, Western Cape, Koeberg Nature Reserve, 33°37.622'S, 18°24.259'E, 5 September – 3 October 1997, S. van Noort, KO97-M09, Malaise trap, West Coast Strandveld, SAM-HYM-P047477 ♀, ♂ South Africa, Northern Cape, Hantam National Botanical Garden, 31°23.802'S, 19°08.799'E, 752m, 23 July 2008 - 23 August 2008, S. van Noort, GL07-REN1-M43, Malaise trap, Nieuwoudtville Renosterveld Shale, SAM-HYM-P044547 (SAMC, BMNH).

**Figure 9.**
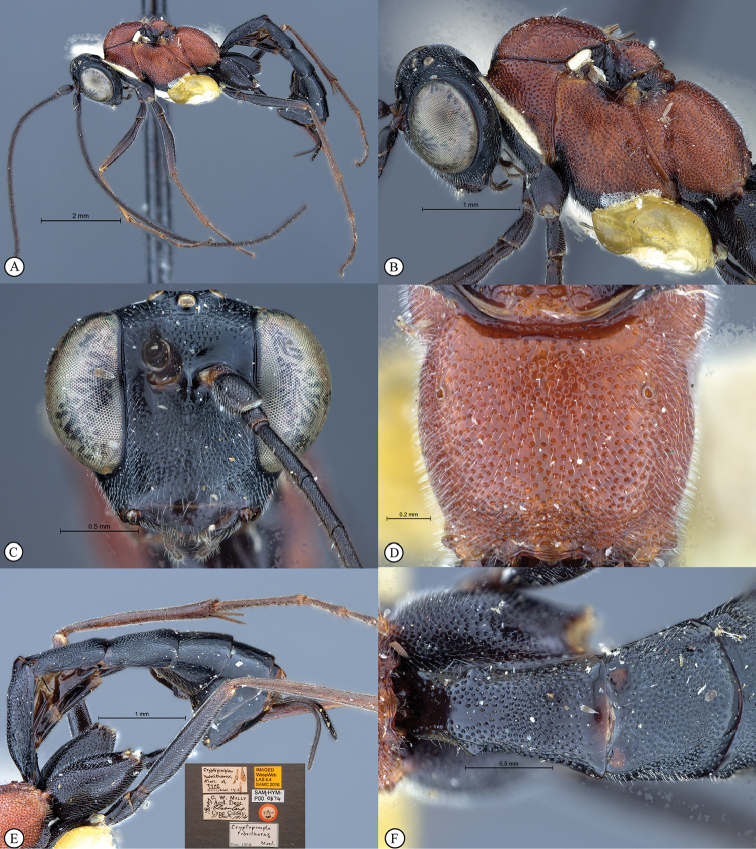
*Cryptopimpla rubrithorax*. Holotype **A** Habitus, lateral view **B** Head and mesosoma, lateral view **C** Head, anterior view **D** Propodeum, dorsal view **E** Metasoma, lateral view inset: data labels **F** Metasomal terga 1 and 2, dorsal view.

##### Description

(updated from Morley, 1916). Body subpolished. Colour. Head and metasoma black, posterior margins of terga 6–8 white. Clypeus rarely distinguished by colour to the rest of the face. Mesosoma rufescent, black ventrally; mesonotum black at the wing bases.

Head. Densely punctate. Frons unarmed. Setae on head and clypeus short and sparse. Clypeus profile weakly convex with a curved lip on the ventral margin. Clypeus edge convex. Upper tooth of mandible longer than the lower tooth. Flagellum tapered to a slender apex. Tentorial pits small or indistinct. Maximum eye width in anterior view 0.6–0.66 shortest inter-ocular distance, eye large in lateral view with maximum width 0.7–0.75 times maximum length.

Mesosoma. Mesosocutum moderately punctate. Scuto-scutellar groove broad with deep lateral indentations. Epicnemial carinae present ventrally and dorsally, dorsally converging toward anterior edge of mesopleuron. Propodeum lacking carinae, its anterior margin medially straight but may have a blunt medial projection. Wings hyaline. Fore wing with two bullae closely situated appearing as one; vein 2m-cu sinuate; areolet truncate-shaped. Hind wing with one basal hamulus and six distal hamuli.

Metasoma. Tergum 1 densely punctate with dorsolateral carinae substituted with longitudinal wrinkles, posterior margin weakly convex; second tergum 0.8–1.09 times as broad as long, spiracle situated at basal 0.25–0.32 of tergum (measured in lateral view), gastrocoeli elongate; terga 4–8 slightly compressed; female metasomal tergum 6 half as wide as tergum 5; hypopygium strongly sclerotized. Ovipositor straight or slightly upcurved; sheath striations present.

CT 1.9–2.2; ML 0.9–1.3; IO 1.9–2.4; OO 1.4–2.1; Fl_1_ 4.3–5.4; OT 0.6; body length 7–8.6 mm; antenna length 7.9–9.7 mm; fore wing length 6.3–6.9 mm.

##### Differential diagnosis.

Reduction of the dorsolateral carinae to longitudinal wrinkles on the first metasomal tergum distinguishes this species from the closely-related species *Cryptopimpla fernkloofensis* and *Cryptopimpla neili*. Elongate gastrocoeli on tergum 2 separate the species from the closely-related species *Cryptopimpla fernkloofensis*, *Cryptopimpla elongatus*, *Cryptopimpla hantami*, and *Cryptopimpla neili*. The malar space and basal mandibular width are more or less equal in length with the malar space 0.91–1.3 times as long as the basal mandibular width, as opposed to the malar length index being much shorter in the closest related species *Cryptopimpla zwarti*, where the malar space is 0.6 times as long as the basal mandibular width. The shortened malar space in *Cryptopimpla zwarti* produces a more globular head shape, compared to a more lenticular head shape in *Cryptopimpla rubrithorax* due to the longer malar space. *Cryptopimpla rubrithorax* can be further separated from *Cryptopimpla zwarti* by the length of tergum 2 relative to its width. In *Cryptopimpla rubrithorax* tergum 2 is 0.8–1.09 times broader than long compared to 1.25 times as broad as long in *Cryptopimpla zwarti*.

##### Etymology.

The species epithet is likely to refer to the rufescent colour of the metasoma of this species (Morley 1916).

##### Distribution.

South Africa (Northern and Western Cape).

##### Comments.

This species occurs in three vegetation types, Strandveld, Mesic Mountain Fynbos and Renosterveld, and exhibits corresponding intra-specific variation in terms of colouration. The specimen sampled from mesic mountain fynbos, which has a white pronotal collar and tegula as per Morley's original description, whereas the specimens from the Renosterveld have a black pronotal collar and tegula. Molecular sequencing demonstrated that there is no genetic divergence between specimens associated with the two different habitats (0% sequence divergence for COI, 28S, and 18S), with two site changes on the COI gene sequence (Reynolds Berry, Matthee and van Noort unpubl. data). Strandveld specimens also have black pronotal collars and tegulae, but are slightly darker in colour and are blacker ventrally on the mesosoma.

#### Cryptopimpla zwarti

Taxon classificationAnimaliaHymenopteraIchneumonidae

Reynolds Berry & van Noort sp. n.

http://zoobank.org/78719B96-6099-49F3-BADA-39E6ABD03C98

Fig. 10

##### Type material.

**HOLOTYPE** ♀: South Africa, Eastern Cape, Grahamstown, Faraway Farm 33.19'S, 19°26.31'E, April 1990, I. Crampton, Malaise trap, SAM-HYM-P005220 (SAMC).

##### Description.

Body subpolished. Colour. Head black, clypeus and mouthparts brown. Mesosoma rufescent, small black spot on underside; mesoscutum black only at the wing bases. Front legs: mostly light brown; coxa, trochanter and trochantellus black. Middle and hind legs: coxa to femora mostly black with shades of light brown on the coxa; remaining parts of leg light brown. Metasoma black, terga 6–8 posteriorly white.

Head. Densely punctate. Frons unarmed. Clypeus profile weakly convex with a curved lip on the ventral margin. Clypeus edge convex. Upper tooth of mandible longer than the lower tooth. Setae on head and clypeus short and sparse. Tentorial pits small and indistinct. Eye in lateral view 0.71 times as wide as long, maximum width in anterior view 0.66 times shortest inter-ocular distance. Flagellum tapered to a slender apex.

Mesosoma. Mesosocutum moderately punctate. Broad scuto-scutellar groove with deep lateral indentations. Epicnemial carinae present ventrally and dorsally, dorsally converging toward anterior edge of mesopleuron. Propodeum without carinae, its anterior margin with a blunt median projection. Wings hyaline. Fore wing with two bullae close together appearing as one; vein 2m-cu sinuate; areolet truncate-shaped. Hind wing with one basal hamulus and six distal hamuli.

Metasoma. Slightly compressed. Tergum 1 with dorsolateral carinae substituted with longitudinal wrinkles, densely punctate with posterior margin weakly convex; second tergum 1.25 times broader than long, spiracle situated at basal 0.3 of tergum, gastrocoeli elongate; tergum 6 half as wide as tergum 5; hypopygium strongly sclerotized. Ovipositor slightly upcurved; sheath striations present.

CT 2; ML 0.6; IO 2.2; OO 1.6 OT 0.6; Fl_1_ 4.9; body length 8.3 mm; antenna length 9.4 mm; fore wing length 6.9 mm.

**Figure 10.**
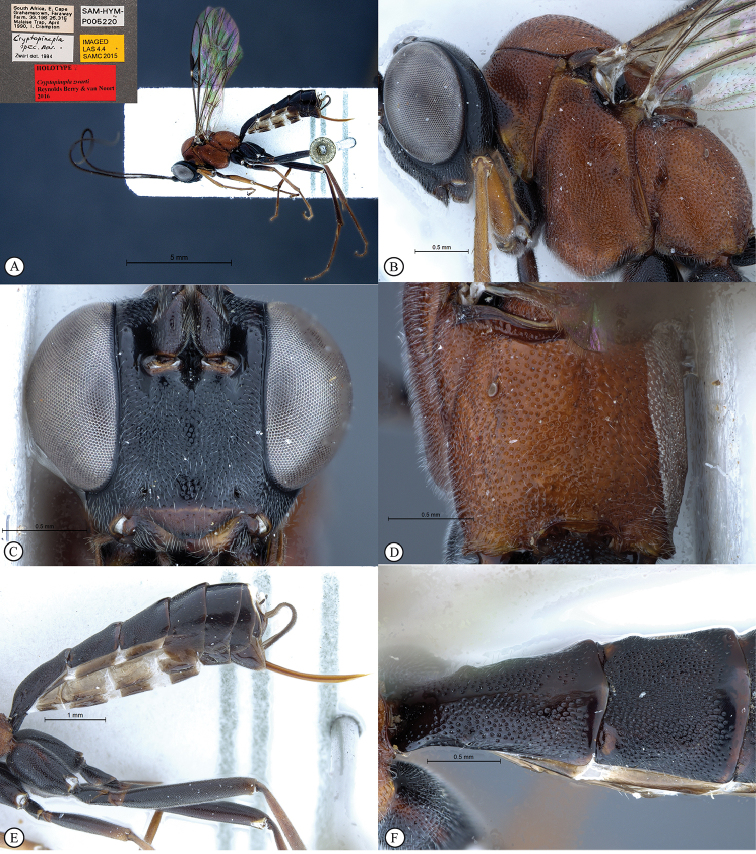
*Cryptopimpla zwarti*. Holotype **A** Habitus, lateral view inset: data labels **B** Head and mesosoma, lateral view **C** Head, anterior view **D** Propodeum, dorsal view **E**) Metasoma, lateral view **F** Metasomal terga 1 and 2, dorsal view.

##### Differential diagnosis.

*Cryptopimpla zwarti* is distinguishable from all other Afrotropical *Cryptopimpla* species by having a malar space 0.6 times as long as the basal mandibular width, whereas all the other Afrotropical *Cryptopimpla* species have a ML index of 0.8 or more. A broad scuto-scutellar groove with deep lateral indentations distinguishes *Cryptopimpla zwarti* from closely-related species *Cryptopimpla fernkloofensis*, *Cryptopimpla neili*, *Cryptopimpla hantami*, and *Cryptopimpla parslactis*. The metasomal tergum 1 with dorsolateral carinae substituted with longitudinal wrinkles distinguishes *Cryptopimpla zwarti* from closely-related species *Cryptopimpla fernkloofensis* and *Cryptopimpla neili*. Gastrocoeli on tergum 2 are elongate separating the species from closely-related species *Cryptopimpla fernkloofensis*, *Cryptopimpla elongatus*, and *Cryptopimpla hantami*.

##### Etymology.

Named after the retired agricultural entomologist, K. W. Robert Zwart (Wageningen Agricultural University) who first recognized it as a potentially new species in 1994. Noun in the genitive case.

##### Distribution.

South Africa (Eastern Cape).

##### Comments.

By having a malar space much shorter than the basal mandibular width (malar index of 0.6), the shape of the head is more globular, which in combination with a second tergum that is broader than long, separates *Cryptopimpla zwarti* from its closely-related species *Cryptopimpla rubrithorax*, which have a malar space 0.91–1.3 times as long as the basal mandibular width, creating a more lenticular-shaped head, and a second tergum that is 0.92–1.2 times as long as broad (i.e. no more than 1.09 times broader than long).

## Discussion

The review of *Cryptopimpla* in the Afrotropical region allowed for a comparative morphological assessment of these species with *Cryptopimpla* species from other biogeographical regions, highlighting differences in regional suites of character states. The presence of a posterior transverse carina on the propodeum is common in world *Cryptopimpla* species (Townes 1969, Sheng 2011), but usually absent in Afrotropical species, only being present in two of the ten known species. Among the few *Cryptopimpla* species outside of the Afrotropical region that do not possess pleural carinae or a posterior transverse carina are *Cryptopimpla labralis* and *Cryptopimpla escarinata* from North America (Townes 1978). *Cryptopimpla labralis*, like species within the *rubrithorax* species-group, possesses a weakly convex clypeus. *Cryptopimpla labralis* is distinctly different from species within the *rubrithorax* species-group by having an areolet that is petiolate, the fore wing lengths are 4.3–4.7 mm long and the OO index is 0.42, compared to the *rubrithorax* species-group where the areolet is truncate-shaped, forewing lengths are 5.8–7.2 mm long and the OO index is 0.5–0.6. Unfortunately, the character state for absence/presence of the posterior transverse carinae on the propodeum is not detailed in all of the historical species descriptions and we have not been able to obtain the types to confirm the state of this condition for most of the global species. It is clear, however, that the lack of carinae on the propodeum and a weakly convex clypeus, are character states that are not restricted to African *Cryptopimpla* species assemblages. This should not compromise the taxonomic results since almost all species of *Cryptopimpla* are exclusive of a single biogeographical region (Yu et al. 2012).

Our morphological species-group delimitation is supported by molecular results based on the mitochondrial COI gene (~23% sequences divergence) and the nuclear 28S gene (~4% sequence divergence). Strong support for monophyly of the genus was obtained from a supermatrix analysis using both molecular (18S, 28S, COI) and morphological data (Reynolds Berry, Matthee & van Noort, unpubl. data).

The species *Cryptopimpla fernkloofensis*, *Cryptopimpla goci*, *Cryptopimpla neili*, and *Cryptopimpla parslactis* are described based on a single male specimen. Without the presence of the diagnostic female character of a shortened ovipositor sheath, male *Cryptopimpla* are sometimes confused and incorrectly described as the cosmopolitan banchine genus *Lissonota* (e.g. Gravenhorst 1829, Holmgren 1860). There are, however, a number of morphological characters that can differentiate *Cryptopimpla* from *Lissonota* when only males are available. The apical 0.3–0.4 portion of the flagellum is tapered to a slender apex in *Cryptopimpla* (Townes 1969, Takasuka et al. 2011) whereas the flagellum is not tapered or may be only weakly tapered at the apex in *Lissonota* (Townes 1969). The first metasomal tergum is only moderately narrowed toward the base in *Lissonota* species as opposed to being evenly and rather strongly narrowed toward the base in *Cryptopimpla* species. Lastly, in all *Cryptopimpla* species the upper tooth is distinctly longer than the lower (Townes 1969; Sheng 2011; Takasuka et al. 2011), whereas it is not a consistent character state for *Lissonota*. While these three character states are useful in distinguishing *Cryptopimpla* from *Lissonota*, our placement of *Cryptopimpla fernkloofensis*, *Cryptopimpla goci*, *Cryptopimpla neili*, and *Cryptopimpla parslactis* in the genus *Cryptopimpla* is further supported by their overall morphological resemblance to females of their respective species-groups.

*Cryptopimpla* is only known from South Africa in the Afrotropical region. The genus was previously represented by a single species in the region and the present study has yielded an additional nine species endemic to temperate areas of South Africa. This is unlikely to be a sampling artifact, given the numerous sampling inventories carried out in other African countries, and the absence of additional specimens in international museum collections. *Cryptopimpla* is a predominately northern hemisphere genus, with highest species richness in the temperate regions (Yu et al. 2012; Sheng and Zheng 2005; Kuslitzky 2007; Sheng 2011; Takasuka et al. 2011). This may explain its apparent exclusion from the tropical regions of Africa. Although the winter rainfall Cape region of South Africa has been fairly extensively sampled over the past 25 years, with deployment of numerous long-term (spanning 1 to 5 years) inventory surveys, ensuring that seasonal variation in species assemblages is encompassed, in effect this effort has only just started scratching the surface with regard to documenting the ichneumonid diversity present in the area. In reality these inventory sites are relatively few and widely spaced, with the implication that the vast majority of the 440 vegetation types (Mucina and Rutherford 2011) in South Africa, Lesotho and Swaziland are still not comprehensively sampled. This fact in combination with the rarity of the genus (only 33 specimens known for the 9 species), with species often represented by a single specimen, suggests that there are still numerous *Cryptopimpla* species to be discovered in South Africa. The current revision has increased the knowledge of African species ninefold and that of the global fauna by ~16%. Further comprehensive sampling will undoubtedly elevate *Cryptopimpla* species richness for the Afrotropical region.

## Supplementary Material

XML Treatment for Cryptopimpla

XML Treatment for Cryptopimpla elongatus

XML Treatment for Cryptopimpla fernkloofensis

XML Treatment for Cryptopimpla goci

XML Treatment for Cryptopimpla hantami

XML Treatment for Cryptopimpla kogelbergensis

XML Treatment for Cryptopimpla neili

XML Treatment for Cryptopimpla onyxi

XML Treatment for Cryptopimpla parslactis

XML Treatment for Cryptopimpla rubrithorax

XML Treatment for Cryptopimpla zwarti
